# Rainfall trends and variation in the Maasai Mara ecosystem and their implications for animal population and biodiversity dynamics

**DOI:** 10.1371/journal.pone.0202814

**Published:** 2018-09-19

**Authors:** Gundula S. Bartzke, Joseph O. Ogutu, Sabyasachi Mukhopadhyay, Devolent Mtui, Holly T. Dublin, Hans-Peter Piepho

**Affiliations:** 1 Biostatistics Unit, Institute of Crop Science, University of Hohenheim, Stuttgart, Germany; 2 Directorate of Research, Tanzania Wildlife Research Institute, Arusha, Tanzania; 3 Wasaa Conservation Centre, IUCN Eastern and Southern Africa Regional Office, Nairobi, Kenya; Fred Hutchinson Cancer Research Center, UNITED STATES

## Abstract

Rainfall exerts a controlling influence on the availability and quality of vegetation and surface water for herbivores in African terrestrial ecosystems. We analyse temporal trends and variation in rainfall in the Maasai Mara ecosystem of East Africa and infer their implications for animal population and biodiversity dynamics. The data originated from 15 rain gauges in the Mara region (1965–2015) and one station in Narok Town (1913–2015), in Kenya’s Narok County. This is the first comprehensive and most detailed analysis of changes in rainfall in the region of its kind. Our results do not support the current predictions of the International Panel of Climate Change (IPCC) of very likely increases of rainfall over parts of Eastern Africa. The dry season rainfall component increased during 1935–2015 but annual rainfall decreased during 1962–2015 in Narok Town. Monthly rainfall was more stable and higher in the Mara than in Narok Town, likely because the Mara lies closer to the high-precipitation areas along the shores of Lake Victoria. Predominantly deterministic and persistent inter-annual cycles and extremely stable seasonal rainfall oscillations characterize rainfall in the Mara and Narok regions. The frequency of severe droughts increased and floods intensified in the Mara but droughts became less frequent and less severe in Narok Town. The timings of extreme droughts and floods coincided with significant periodicity in rainfall oscillations, implicating strong influences of global atmospheric and oceanic circulation patterns on regional rainfall variability. These changing rainfall patterns have implications for animal population dynamics. The increase in dry season rainfall during 1935–2015 possibly counterbalanced the impacts of resource scarcity generated by the declining annual rainfall during 1965–2015 in Narok Town. However, the increasing rainfall extremes in the Mara can be expected to create conditions conducive to outbreaks of infectious animal diseases and reduced vegetation quality for herbivores, particularly when droughts and floods persist over multiple years. The more extreme wet season rainfall may also alter herbivore space use, including migration patterns.

## Introduction

A better understanding of rainfall dynamics is indispensable for developing biodiversity conservation measures likely to be effective under climate change [1]. Such understanding requires carefully verified observational data to ensure accuracy and reliability. This is especially pertinent for Africa where high-quality observational rainfall datasets with sufficiently high spatial and temporal resolutions are rare [2] and noteworthy discrepancies often exist between digital datasets and original weather records [3] even for the same weather stations [4].

Here, we use carefully verified station rainfall data for the Maasai Mara ecosystem to answer the following questions. (1) Are there temporal trends in the monthly, annual and seasonal rainfall components? (2) Are there shifts in rainfall seasonality? (3) What are the dominant cycle periods of oscillations in the rainfall components and are the periods changing? (4) Are severe droughts and floods becoming more frequent and severe and do they persist over multiple years? (5) How might the changing rainfall patterns affect animal population and biodiversity dynamics based on known responses of animal abundance, reproduction, survival, disease susceptibility and migration to rainfall?

Rainfall is the principal driver of the population dynamics of savanna herbivores [5,6] because it controls plant biomass production [7,8] and plant nutrient concentration [9], which affect herbivore birth [6] and survival [10] rates, susceptibility to predation [11] and, ultimately, biomass [12,13]. Not surprisingly, oscillatory dynamics in ungulate population size [5] and ungulate fecundity [14] are coupled with inter-annual and seasonal rainfall oscillations in African savannas, respectively.

Droughts can cause substantial herbivore mortality and often regulate population size [15]. For example, 75% of wildebeest (*Connochaetes taurinus mearnsi* (Burchell)) deaths in Serengeti were caused by undernutrition and rainfall was the most important factor determining food supply [16]. Concentrations of herbivores around water points during droughts [17] can elevate vegetation damage [18], and result in increased competition and predation [19].

Droughts and floods also facilitate infestations by parasites [20] and diseases such as the canine distemper virus outbreak among Serengeti lions following a severe drought in 1993 [21]. Excessive rainfall can adversely affect small herbivores that require high-quality forage through diluting plant nutrient concentration [22]. High rainfall also promotes fires because it increases fuel loads in grasslands [23]. But low rainfall can lead to more destructive and extensive fires in savanna woodlands and forests [24].

Rainfall distribution and seasonality principally drives animal migration [25] and dispersal [26] in savannas. Consequently, animals may alter both their migratory [25] and short-term movements rapidly in response to localised rainfall patterns. During low rainfall years, animals are forced to travel longer distances between water and foraging grounds, making their offspring more vulnerable to predation [27].

Temperatures have risen in recent decades in most parts of the eastern African region [2,28–30] but the contemporaneous changes in rainfall seem subtle and largely unpredictable [2]. Rainfall increased in parts of East Africa during 1951–2001 [31] and during 1979–2010 [2]. Thus, rainfall trends around Lake Victoria were predominantly positive over the 20^th^ century [32]. Likewise, General Circulation Models project increasing rainfall [33–36], more intense wet seasons and less severe droughts for most of East Africa [37]. Climate models also project strengthening of the El Niño Southern Oscillation (ENSO) and more frequent occurrences of the positive phase of the Indian Ocean Dipole [38,39] as temperatures rise, although evidence for the strengthening of the ENSO phenomenon remains controversial [40,41]. Such conditions facilitate moisture export from the Indian Ocean towards East Africa by weakening westerly winds [42,43] and lead to more intense wet seasons and floods.

The above patterns are in contrast to the findings of other studies of temporal trends in East African rainfall, such as decreasing annual [30,33], wet season [44–46] and dry season rainfall [36] in recent decades. Concurrently, droughts became more severe during 1970–2006 in Eastern and Southern Africa [44]. La Niña events, which often follow extreme El Niño events [47], typically lead to severe drying in East Africa [48]. Rainfall also declines during negative phases of the Indian Ocean Dipole [49]. For example, the 2005–2006 East African drought was associated with both a strong negative Indian Ocean Dipole and La Niña-like conditions [50,51].

Contrasting rainfall trends have also been documented for the Mara-Serengeti ecosystem. Ritchie et al. [52] reported a decrease in the total annual and wet season rainfall during 1960–2001 but an increase in the dry season rainfall in the Serengeti during 1913–2001. In contrast, Ogutu et al. [53] reported a decline in the dry season rainfall in the Maasai Mara Reserve (Mara Reserve) during 1975–2003. However, dry season rainfall in Narok, a Kenyan town located 75 km north-east of the Mara Reserve, increased during 1940–2004 after a protracted drought during 1930–1939 [53]. These contrasting findings demonstrate considerable uncertainty inherent in trends and variation in the past and anticipated future rainfall scenarios [35].

Climate warming can change rainfall seasonality and cycle periods by modulating ocean-atmosphere circulations. Climate warming alters ocean temperatures, cloud and ice cover, leading to shifts in the movement of the Inter-Tropical Convergence Zone [54]. This belt of rising and convecting air masses driven by solar radiation is the causal agent for rainfall seasonality as it moves southwards from East Africa during the transition between the dry and the wet season (July-January) and northwards during the transition between the wet and the dry season (January-July) [55].

The dominant East African rainfall cycle periods ranging between about 2 to 12.5 years appear very variable in space and time [56–58]. Besides the ENSO phenomenon and the Indian Ocean Dipole, oscillations in Atlantic ocean temperatures can influence East African rainfall considerably [59] through its teleconnections (an influence occurring over large distances, typically thousands of kilometres) via the Indian Ocean [60] and the West African monsoon [61]. The highly variable local topography of the African Rift Valley may also contribute to temporal and spatial variability in rainfall [62].

The aim of this study was to quantify trends and variation in rainfall in the Maasai Mara ecosystem in East Africa as a background for understanding their past and possible future implications for animal population and biodiversity dynamics.

## Materials and methods

### Study area

**Fig 1:**
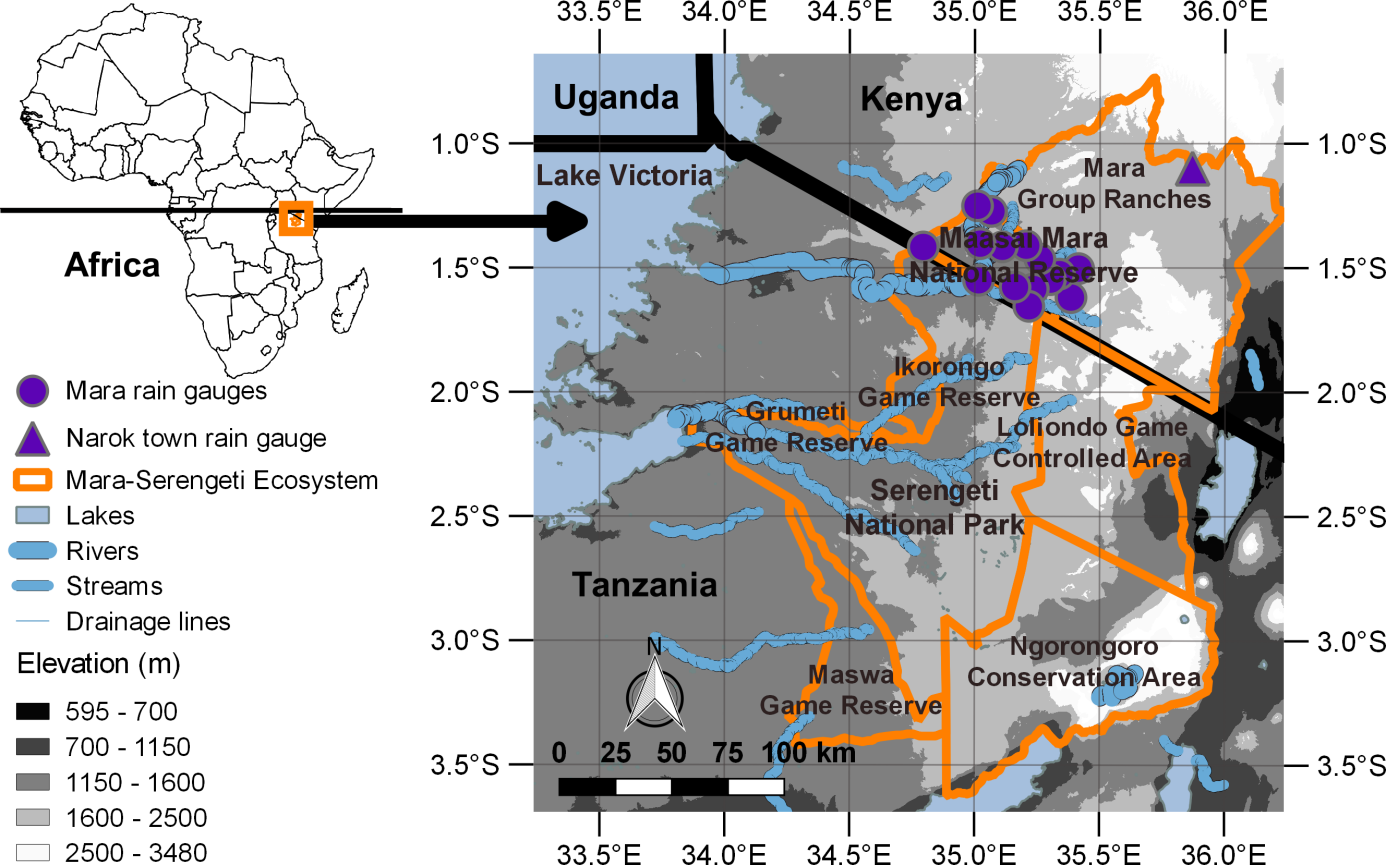
The Mara-Serengeti ecosystem (orange borders) straddling the international border (black line) between Tanzania and Kenya.

The Maasai Mara ecosystem is situated along the international border between Kenya and Tanzania in equatorial East Africa (34.7° to 35.4° E, 1.2° to 1.7° S, Fig 1). The elevation in the Mara Reserve ranges from about 1,450 m to about 2,100 m above sea level (Fig 1). The Mara Reserve was established in 1961 and the adjoining Serengeti National Park in 1951 to protect the rainfall-driven migration of the numerous wildebeest, zebra (*Equus quagga burchellii*) and Thomson’s gazelle (*Gazella thomsonii*) [63]. Multiple buffer zones with various degrees of protection and land use types now surround the protected areas [9] (Fig 1). The Maasai Mara ecosystem of Kenya consists mostly of grasslands with the cover of shrubs and thorny bushes increasing towards its northern and eastern extremes [64]. The dominant vegetation type in the Serengeti is savanna with a mixture of grasses, shrubs and trees [63]. Riverine forests fringe various streams and drainage lines in the area [63] (Fig 1) but much of this forest has been lost.

The entire Mara-Serengeti ecosystem supports an extremely diverse and abundant community of herbivores [65] and carnivores [66]. Populations of many wildlife species are declining, concurrent with changes in climate, growing pressures from cultivation, livestock grazing and other anthropocentric influences that are driven by accelerating human population growth [30,63,67]. The major land use type in the Maasai Mara ecosystem is pastoralism [30]. Higher rainfall areas in the buffer zone more distant from the Mara Reserve are now largely converted into agriculture [64]. Settlements, extensive agriculture and sedentary livestock holdings characterize the western side of the Serengeti National Park today [63].

The climatic year in the Mara starts in November and ends in October of the following year. The seasonal rainfall distribution is strongly bimodal, with the wet season spanning November-June and the dry season spanning July-October. The wet season consists of the short (November-December) and the long (January-June) rains. January-February trends to be dry and hence is sometimes called the short dry season in contrast to the long dry season from July-October [68]. The annual rainfall in the Mara reserve follows a spatial continuum from about 650 mm in the southeast to about 1300 mm in the northwest [68]. The dry season rainfall in particular is higher and more stable close to Lake Victoria ([55], Fig 1), the largest lake in Africa. Climate models suggest that the difference in air temperature between the land and the lake water creates a local convergence zone [69] that interferes with large-scale atmospheric and oceanic circulation patterns [70] and brings rain to the north-western extensions of the Mara-Serengeti ecosystem [55]. Consequently, rainfall decreases away from Lake Victoria, reaching about 750 mm in Narok Town in Kenya and 350–450 mm on the south-eastern Serengeti plains in Tanzania [63].

During the dry season, the seasonal watercourses dry out and the remaining isolated pools, ponds, springs and the Mara River, the only permanent river in the Mara-Serengeti ecosystem, serve as the only sources of water for wildlife and livestock across the area [71]. As water levels drop, the water quality of these remaining sources becomes very poor, in part because wildlife concentrate near water as the dry season progresses [71]. Forage quality and quantity progressively decline with time after the wet season [72]. Consequently, wildebeest migrate back from the south-eastern Serengeti Plains to the Mara in Kenya where the dry season rainfall is higher [55]. Poor rainfall can force wildebeest to leave these nutrient-rich grass plains in Serengeti earlier than usual but increased rainfall can cause them to return earlier [25].

Direct correlations between rainfall and ENSO appear weak in the Mara-Serengeti ecosystem [53,73] but major El Niño events can cause substantial mortality of herbivores [74]. Similarly, La Niña events [50,51] are sometimes followed by marked reductions in herbivore biomass [75]. The dominant rainfall cycles in the Mara-Serengeti ecosystem have periods of about 5 to 10 years but cycle lengths can vary widely [76].

In addition to rainfall variability, water flow in the Mara River has been declining as a consequence of upstream deforestation of the Mau forest and excessive water abstraction for irrigation in Kenya [77].

### Data sources and processing

We obtained total monthly rainfall data for 15 gauges in the Mara spanning 1965–2014 (S1 Data) and for one gauge for Narok Town spanning 1913–2015 (S2 Data) from the sources listed in Table 1. Thirteen of the 15 gauges in the Mara were operated by the World Wide Fund for Nature (WWF) and Friends of Conservation (FOC) as part of the Maasai Mara Ecological Monitoring Programme from 1989 to 2003. The records for 5 of the 15 gauges were taken daily and then summed to obtain monthly totals. Rainfall for the other gauges was measured at monthly intervals.

**Table 1:** The sources of rainfall records for 15 rain gauges in the Mara and a rain gauge in Narok Town in Kenya.

Station	Coordinates^a^		Elevation (m)	Period (year-month)		Frequency	% Missing monthly	% Missing daily
	Eastings	Northings		Start	End			
**Narok Town**^**b**^	819064	9878264	1,869	1913–04	2015–12	Daily	<1	n.a.
**Keekorok Hydromet Station**^**b**^	748557	9824984	1,634	1965–01	1997–11	Daily	7	n.a.
**Hyena Camp**^**c**^	751839	9837939	1,585	1988–08	2015–03	Daily	0	1
**Sekenani Gate**^**d**^	760229	9831568	1,740	1989–05	1996–03	Daily	5	n.a.
**New Mara Bridge**^**d**^	724211	9828830	1,487	1989–06	2003–12	Monthly	9	n.a.
**Ngiro-Are Ranger Station**^**d**^	699634	9843664	1,626	1989–06	2003–12	Monthly	30	n.a.
**Ololaimutia Gate**^**d**^	765333	9821144	1,828	1989–06	2003–12	Monthly	6	n.a.
**Roan Hill**^**d**^	740442	9825830	1,596	1989–06	2003–12	Monthly	9	n.a.
**Sand River Gate**^**d**^	746575	9817278	1,608	1989–06	2003–12	Monthly	6	n.a.
**Cottars Camp/Siana Springs**^**d**^	768714	9834029	1,745	1989–12	2003–12	Monthly	7	n.a.
**Musiara Gate**^**d**^	729954	9859508	1,567	1989–12	2003–12	Monthly	9	n.a.
**Mara Research Station**^**d**^	756181	9829120	1,781	1989–12	2003–12	Monthly	6	n.a.
**Mara Intrepids**^**d**^	734780	9843734	1,521	1990–01	1995–08	Daily	4	n.a.
**Kichwa Tembo**^**d**^	723768	9862008	1,658	1990–01	2003–12	Monthly	8	n.a.
**Serena Lodge**^**e**^	724926	9845143	1,600	1990–01	2003–12	Daily	8	n.a.
	724390	9845214	1,584	2008–07	2013–12	Daily	0	1
**Talek Gate**^**d**^	745465	9840275	1,561	1992–07	2003–12	Monthly	9	n.a.

n.a., not available.

^a^Coordinates are given in UTM, Zone 36 S, WGS 84.

^b^Source: Kenya Meteorological Department

^c^Source: Professor Kay E. Holekamp

^d^Source: Maasai Mara Ecological Monitoring Programme

^e^Source: Maasai Mara Ecological Monitoring Programme (1990–2003) and Professor Kay E. Holekamp (2008–2013)

Due to some obvious inconsistencies and discrepancies in the rainfall data sets supplied by government institutions, administrative bodies, research institutions and individual researchers [4], the Kenya Meteorological Department verified 93% of the monthly values for Narok Town Meteorological Station and 97% of the monthly rainfall values for Keekorok Hydromet Station availed to us against the original records that were handwritten either on data cards or in ledger books. When data cards were not available, we used the values recorded in the ledger books or other digital files. Thus we were able to compile, in our estimation, the most accurate historical rainfall dataset available for the study region for 1913–2015. For periods when no records were available for any of the gauges in the Mara (e.g. March 1968 to April 1969) we imputed total monthly rainfall values using the state space method of Piepho and Ogutu [78]. Only one missing monthly record (January 2014) was imputed for Narok Town.

We derived a single time series of rainfall across all available gauges in the Mara after adjusting monthly rainfall by the mean monthly rainfall at individual gauges (Eq 3 in S1 Text). This method performed best among four different standardisation approaches considered for accounting for spatial variation in rainfall in the Mara region (S1 Text). The standardisation methods (S1 Fig) were assessed in terms of how well they accounted for the level shift in rainfall due to the closure of several recording stations after 2003 and whether they reproduced the well-documented incidences of extreme events in the Mara-Serengeti ecosystem such as the extreme floods of 1998, the extreme droughts of 1982 and 2006, and the severe droughts of 1984, 1993, 1999, 2000 and 2009 [29,75,79–82].

**Fig 2:**
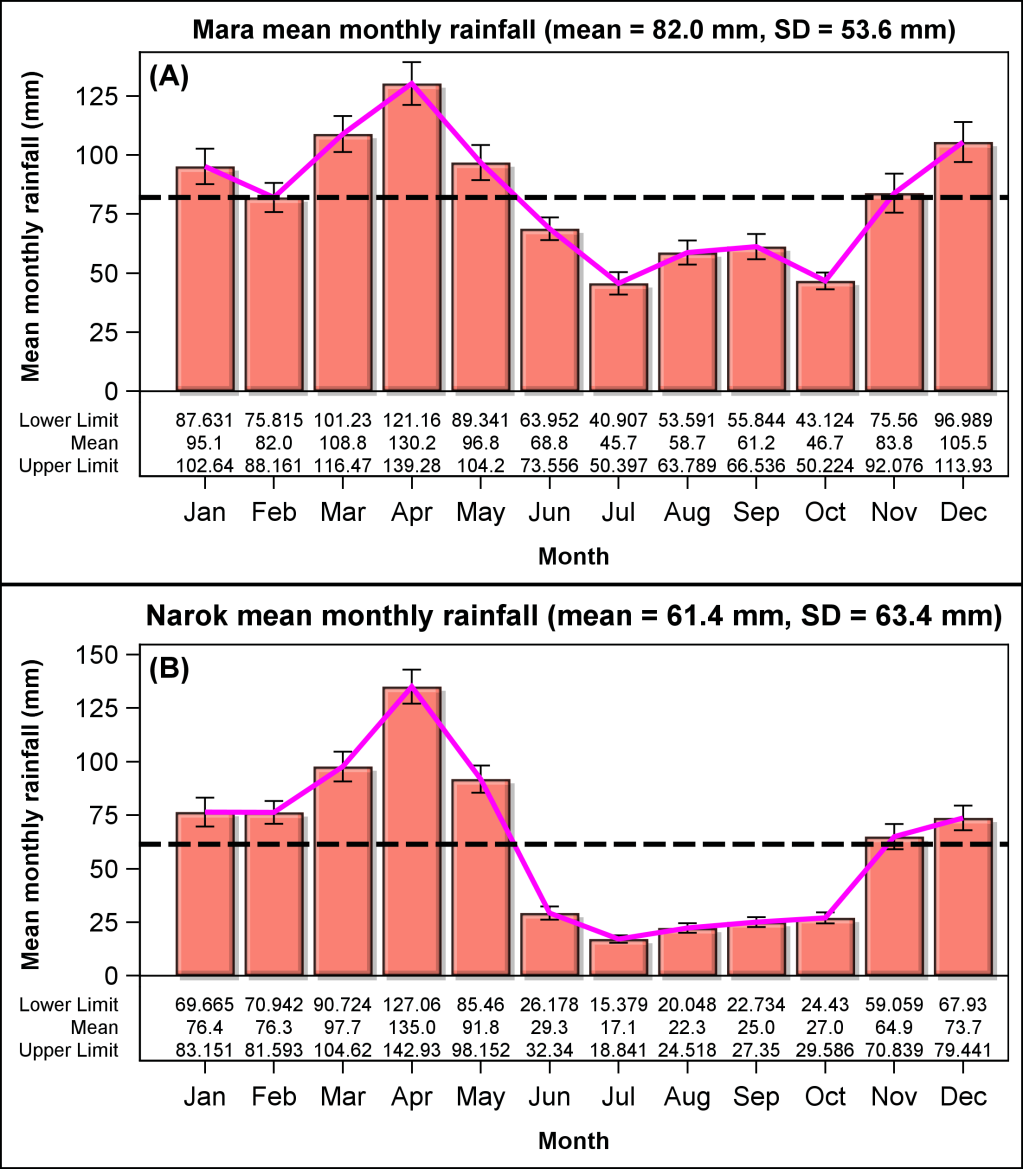
The distribution of total monthly rainfall across months. (A) Rainfall recordings in the Mara were derived from 15 gauges (Eq 3 in S1 Text) during 1965–2015. (B) Rainfall in Narok Town in Kenya was recorded during 1913–2015.

We summed up the dry season rainfall spanning the months July-October in the Mara (Fig 2A) and June-October in Narok Town (Fig 2B) based on the distribution of monthly rainfall. In June, the rainfall in Narok Town is already much lower than rainfall for the wet season months unlike for the Mara, where migratory wildebeest normally arrive in July to occupy their dry season range [25]. The wet season rainfall spans two consecutive calendar years; November of the preceding year up to June of the current year in the Mara but up to May in Narok Town.

### Statistical analyses

#### Temporal trend and seasonality in monthly rainfall

We used the unobserved components model (UCM, [83]), a special case of the linear Gaussian state space model, that is well suited to simultaneously analyse changes in trends (${\dot{\mu }}_{n}$) and seasonality (*γ*_*n*_) in monthly rainfall levels (*x*_*n*_) while taking account of autocorrelation (${\dot{a}}_{n}$) by decomposing the time series for the *n*-th months as follows:

$$x_{n}={\dot{\mu }}_{n}+\gamma_{n}+{\dot{a}}_{n},\;n=0,1,\ldots ,N-1.$$ (1)

This model assumes statistical independence of the different model components.

We first assume a random walk model for the time trend, or equivalently, that the trend ${\dot{\mu }}_{n}$ remains approximately constant through time. The random walk trend model can be specified for the *n*-th month as

$${\dot{\mu }}_{n}={\dot{\mu }}_{n-1}+{\dot{\eta }}_{n},\;{\dot{\eta }}_{n}∼i.i.d.N(0,\sigma_{\dot{\eta }}^{2}),$$ (2)

in which ${\dot{\eta }}_{n}$ are independent and identically distributed (i.i.d.) normal errors or disturbances having zero mean and variance $\sigma_{\dot{\eta }}^{2}$, assuming that ${\dot{\eta }}_{n}$ is a Gaussian white noise process. It is noteworthy that $\sigma_{\dot{\eta }}^{2}$ = 0 implies that ${\dot{\mu }}_{n}$ is a constant. A significant disturbance (error) variance $\sigma_{\dot{\eta }}^{2}$ implies that the level component ${\dot{\mu }}_{n}$ is stochastic.

Besides the random walk model (2), we modelled the trend component using a locally linear time trend incorporating level and slope components and specified by

$${\dot{\mu }}_{n}={\dot{\mu }}_{n-1}+{\dot{\beta }}_{n-1}+{\dot{\eta }}_{n},\;{\dot{\eta }}_{n}∼i.i.d.N(0,\sigma_{\dot{\eta }}^{2}),$$ (3)

$${\dot{\beta }}_{n}={\dot{\beta }}_{n-1}+{\dot{\xi }}_{n},\;{\dot{\xi }}_{n}∼i.i.d.N(0,\sigma_{\dot{\dot{\xi }}}^{2}),$$ (4)

where the disturbances ${\dot{\eta }}_{n}$ and ${\dot{\xi }}_{n}$ are assumed to be independent. We dropped the locally linear time trend ${\dot{\beta }}_{n}$ when it was non-significant. The seasonal oscillations were modelled with trigonometric components (S2 Text).

#### Temporal trend and variation in the annual and seasonal rainfall components

Analogous to the time series of the total monthly rainfall records we used the unobserved components model to estimate the overall annual, as well as the wet and dry season rainfall trends (*μ*_*t*_) and cycles (*φ*_*t*_) simultaneously by decomposing the time series (*r*_*t*_):

$$r_{t}=\mu_{t}+\varphi_{t}+\epsilon_{t},\;t=0,1,\ldots ,T-1,$$ (5)

in which *ϵ*_*t*_ are i.i.d. normally distributed errors or disturbances having zero mean and variance *σ*^*2*^_*ϵ*_ so that *ϵ*_*t*_ is a Gaussian white noise process. The trend component *μ*_*t*_ is defined analogously to Eq 2, where *η*_*t*_ are now the i.i.d. normally distributed stochastic disturbances.

#### Oscillations in annual and seasonal rainfall components

In addition to the trend components we estimated the periods (*p >* 2) and damping factors (*ρ*) of the stochastic cycle components (*φ*_*t*_) with a time-varying amplitude and phase given by

$$\left[\begin{array}{c} \varphi_{t}\\ \varphi_{t}^{*} \end{array}\right]=\rho \left[\begin{array}{cc} cos\lambda & sin\lambda \\ -sin\lambda & cos\lambda \end{array}\right]\;\left[\begin{array}{c} \varphi_{t-1}\\ \varphi_{t-1}^{*} \end{array}\right]+\left[\begin{array}{c} \upsilon_{t}\\ \upsilon_{t}^{*} \end{array}\right],\;\upsilon_{t},\upsilon_{t}^{*}∼i.i.d.(0,\sigma_{\upsilon }^{2}),$$ (6)

where 0 *< ρ ≤* 1, *λ =* 2*×π/p* is the angular frequency of the cycle with 0 *< λ < π*, *υ*_*t*_ and υ*_t_ are independent Gaussian disturbances with zero mean and variance *σ*^2^_*υ*_. Values of *ρ*, *p* and *σ*^2^_*υ*_ are estimated from the data alongside the other model parameters. Significant stochastic cycle disturbance variances (*σ*^2^_*υ*_) imply stochastic and transient cycles.

The damping factor *ρ* governs the stationarity properties of the random sequence *φ*_*t*_ such that *φ*_*t*_ has a stationary distribution with mean zero and variance *σ*^2^_*υ*_*/*(1 ‒ *ρ*^2^) if *ρ <* 1 but is nonstationary if *ρ =* 1. A damping factor close to one indicates stable and persistent cycles. We specified and tested for significance of up to two cycles in the annual, wet season and dry season rainfall components.

The UCM models (1) and (6) were fitted by the diffuse Kalman filtering and smoothing algorithm [84] in the SAS UCM procedure [83].

#### Analysis of changing periodicity in monthly rainfall oscillations

To reveal temporal changes in the periodicity of monthly rainfall oscillations that are hard to expose using UCM models, we applied wavelet analysis techniques using the R-package biwavelet Version 0.20.10 [85]. For the wavelet analysis, we de-seasonalized the monthly rainfall values by subtracting from each month the average of the total monthly rainfall values for that month. The de-seasonalized values were subsequently transformed with the Morlet wavelet function [86]:

$$\psi_{0}(\kappa )=\pi^{-1/4}e^{i\omega_{0}\kappa }e^{-\kappa^{2}/2},$$ (7)

using Eq 1 in S3 Text where *κ* is a nondimensional “time” parameter and *ω*_0_ is the nondimensional frequency. Significant oscillations in the time series of monthly rainfall were derived using normalized wavelet power spectra (Eq 2 in S3 Text) and plotted.

#### Frequency, severity and timing of severe droughts and floods

We classified drought and flood years or seasons as severe or extreme. We define a year as a severe drought or flood year if the annual or seasonal rainfall component for the year (season) does not reach the estimated 10-year return level, implying that such low rainfall occurs with a probability of less than 0.1 Rainfall does not reach the 20-year return level estimated from the low rainfall values during extreme droughts. We use the term floods to denote very wet years or seasons. A severe flood year is one in which rainfall exceeds the 10-year extreme return level for the annual or seasonal component. However, rainfall exceeds the 20-year return level estimated from the high rainfall values during extreme flood years or seasons. This classification characterizes the extent of rainfall deficit or surfeit and portrays the associated broad transitions in rainfall influences on vegetation production and quality for herbivores.

To find out if droughts and floods are becoming more frequent, we first estimated return levels of droughts and floods for the annual, wet season and dry season rainfall components by applying extreme value analysis using the threshold excess approach in the R-package extRemes [87]. The return level is the estimated amount of rainfall that is not reached or exceeded once within a specified return period. Return levels are comparable to quantiles but are estimated statistically rather than empirically. The generalized Pareto distribution was used to approximate the distribution of extremely low annual and seasonal rainfall components as detailed in S4 Text.

Finally, we assessed if droughts and floods are becoming more frequent by counting their occurrences before and after the midpoints of the annual, wet season and dry season rainfall time series.

#### Multiannual persistence of severe droughts and floods

To detect multiannual persistence of severe droughts and floods, we derived the extremal index based on the runs estimator [88] in the R-package extRemes (Eq 1 in S5 Text). An extremal index smaller than 1 indicates that droughts and floods persist over multiple years. To determine if severe droughts and floods are clustered in time without necessarily persisting over consecutive years, we additionally obtained the extremal index based on the intervals estimator [88] in the R-package extRemes (Eq 2 in S5 Text). Additionally, we tested for non-randomness in the sequence of severe drought and flood years for the annual and seasonal rainfall components using exact runs tests [89] in the R-package randtests version 1.0 [90]. Significant runs tests and values smaller than 1 for the extremal indices indicate that droughts and floods cluster in time.

#### Trends in the severity of droughts and floods

To establish if droughts and floods are becoming more severe, we analysed temporal trends in extremely low (0.05 and 0.10 quantiles), high (0.90 and 0.95 quantiles) and intermediate (0.50 quantile) annual, wet season and dry season rainfall components with quantile regression (S6 Text) using the R-package quantreg version 5.26 [91]. Quantile regression is similar to ordinary linear regression in principle but can use other quantiles than the median, the only quantile handled by ordinary regression assuming normally distributed errors.

We started with an intercept-only null model and sequentially added polynomial terms of increasing order in time (*y*_*t*_). We retained the higher-order terms in the model if they reduced AICc (corrected Akaike Information Criterion) values compared to corresponding models with lower-order term(s) by at least two units [92]. Standard errors of the coefficients were estimated by bootstrapping using the xy-pair method and 10,000 bootstrap replications. The xy-pair method randomly resamples with replacement, pairs of the explanatory (year *y*_*t*_) and response (rainfall *r*_*t*_) variable [93].

The significance threshold was set at 0.05. All analyses (S1 File) were done in R version 3.3.1 [94] except for the UCM models (S2 File) that were implemented in the SAS UCM Procedure, SAS version 9.4 [95], SAS/ETS version 14.1 [83].

## Results

### Temporal trend and variation in rainfall

#### Temporal trend and seasonality in monthly rainfall

The average (mean ± 1sd) total monthly rainfall was higher in the Mara (82 ± 54 mm; Fig 2A) than in Narok Town (61 ± 63 mm; Fig 2B). The level of monthly rainfall remained nearly constant in the Mara during 1965–2015 (S2A Fig) and in Narok Town during 1913–2015 (S2B Fig). There was a slight peak in the level of monthly rainfall around 1963 in Narok Town according to UCM analysis (S2B Fig).

Rainfall was markedly bimodal throughout the study period (Fig 2). There was a minor peak in December during the short rains (November-December) and a major peak in April during the long rains spanning January-June in the Mara (Fig 2A) and January-May in Narok Town (Fig 2B). The dry season spanning July-October in the Mara (Fig 2A) and June-October in Narok Town (Fig 2B) separates the long rains from the subsequent short rains. The average monthly rainfall for the dry season was much lower and more variable for Narok Town (24 ± 25 mm, CV = 104%) than for the Mara (53 ± 34 mm, CV = 64%), even though June was part of the dry season for Narok Town but not for the Mara. The average wet season rainfall was also more variable but only marginally lower for Narok Town (88 ± 69 mm, CV = 78%) than for the Mara (96 ± 56 mm, CV = 58%), despite June being part of the wet season for the Mara but not for Narok Town.

There was no significant shift in rainfall seasonality over the recording period for both the Mara and Narok Town. Rainfall seasonality was strongly deterministic (non-random) and persistent both in the Mara and in Narok Town as indicated by the highly significant seasonal components (S1 Table) that were characterized by highly insignificant disturbance variances (S2 Table). Rainfall seasonality was also remarkably stable based on cumulative averages of the total monthly rainfall for both the Mara (S3A Fig) and Narok Town in Kenya (S3B Fig).

The random sequence of standardized rainfall shows that periods with below-average dry season rainfall frequently received above-average wet season rainfall both in the Mara and in Narok Town (S4 Fig). Conversely, several periods of below-average wet season rainfall were followed by periods of above-average dry season rainfall (S4 Fig).

#### Temporal trend and variation in the annual and seasonal rainfall components

**Fig 3:**
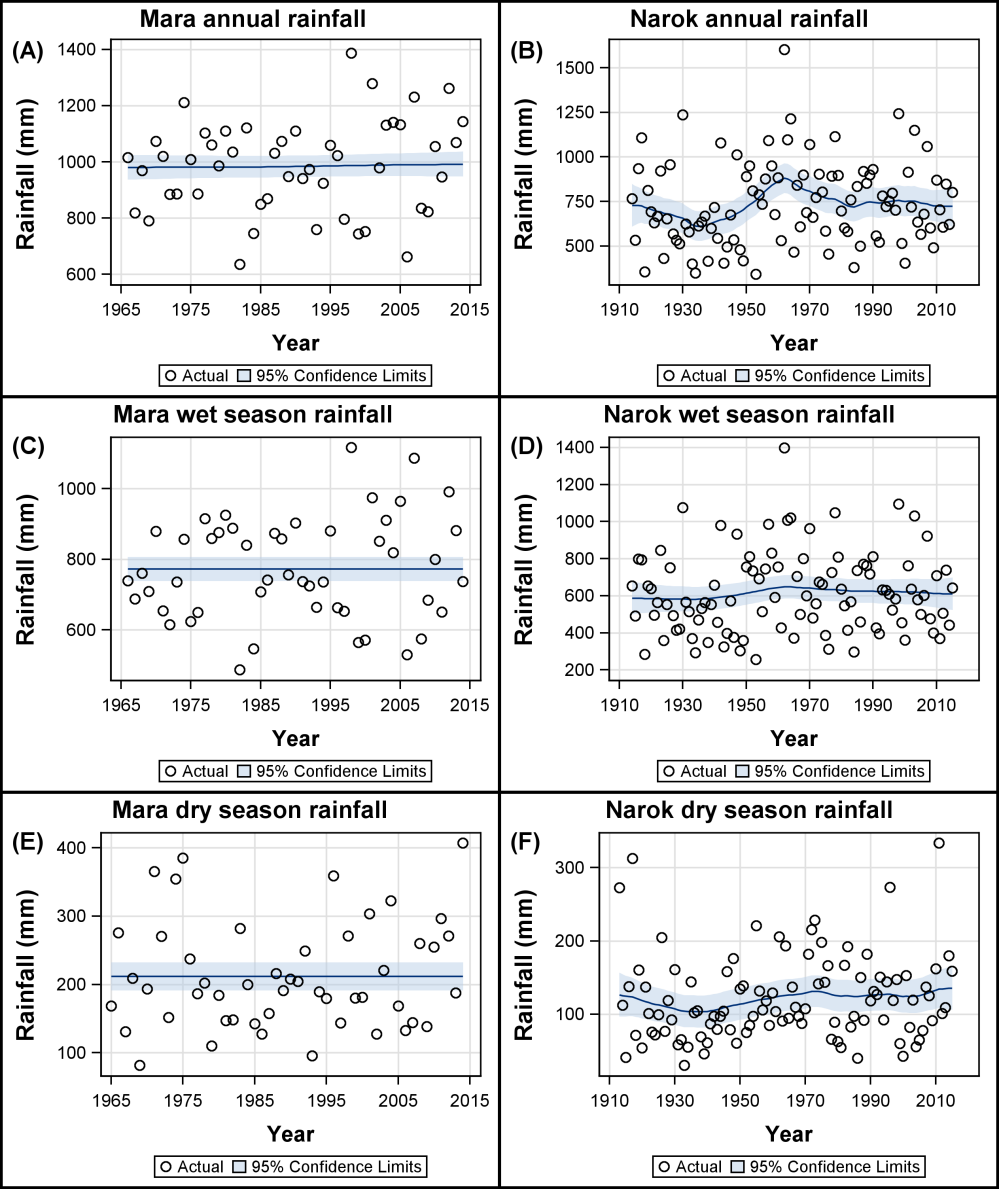
Smoothed level component based on the structural time series analysis of the annual, wet season and dry season rainfall. (A, C, D) Rainfall recordings in the Mara were derived from 15 gauges (Eq 3 in S1 Text) during 1966–2014 (dry season: 1965–2014). (B, D, F) Rainfall in Narok Town in Kenya was recorded during 1914–2015 (dry season: 1913–2015). The (A, B) annual, (C, D) wet season and (E, F) dry season rainfall components were summed from the monthly rainfall records.

There was considerable inter-annual variation in the wet season and dry season rainfall components both in the Mara (S4A Fig) and in Narok Town (S4B Fig). However, only the level components (Eq 5) of the annual (Fig 3B) and the dry season rainfall (Fig 3F) in Narok Town changed notably over time. From 1917 onwards there was a decreasing trend in the annual rainfall component that switched to an increasing trend in 1935, after which rainfall increased from a low of about 600 mm to a high of about 850 mm in 1962 (Fig 3B). Thereafter rainfall declined persistently to about 700 mm in 2015 (Fig 3B). The dry season component initially decreased from about 125 mm in 1913 to about 105 mm in 1940 and then increased to about 135 mm in 2015 (Fig 3F).

### Oscillations in annual and seasonal rainfall components

The rainfall oscillations in the Mara had approximately 3-year cycles for the annual, wet season and dry season rainfall components based on the structural (UCM) time series analyses (Table 2, S5A, S5C and S5E Fig). For Narok Town, the oscillations had approximate 5.2-year cycles for the annual (S5B Fig) and wet season (S5D Fig) rainfall components and 2.3-year cycles for the dry season (S5F Fig) rainfall component (Table 2). The amplitude of the oscillations in the primary wet season cycles became wider during 1995–2015 than during the earlier period 1965–1994 in the Mara (S5C Fig) and was widest during 1950–1965 in Narok Town (S5D Fig). Structural time series analysis also identified high frequency secondary 2.2-year cycles in the annual rainfall component for the Mara (Table 2, S6A Fig) and 2.3-year cycles for Narok Town (Table 2, S6B Fig). Secondary 2.2-year cycles for the Mara (Table 2, S6C Fig) and 2.5-year cycles for Narok Town (Table 2, S6D Fig) were identified for the wet season rainfall component.

**Table 2:** Estimated disturbance variances, damping factors and periods of the cycles in the annual, wet and dry season rainfall components.

			Mara^a^				Narok^b^			
Rainfall component	Model component	Parameter	Estimate	Standard error	t Value	Approx. P-value^c^	Estimate	Standard error	t Value	Approx. P-value^c^
**Annual**	Irregular^d^	Stochastic disturbance (*σ*^2^_*ϵ*_)	15967	3493.5	4.57	<0.0001	0.4994	201.9408	0.00	0.9980
**Annual**	Level	Stochastic disturbance (*σ*^2^_*η*_)	13.2943	98.34848	0.14	0.8925	736.6731	512.4710	1.44	0.1506
**Annual**	Cycle 1^e^	Damping factor (*ρ*)	1.0000	0.0002071	4829.60	<0.0001	0.7595	0.1343	5.66	<0.0001
**Annual**	Cycle 1^e^	Period (*p*)	2.9948	0.02464	121.53	<0.0001	5.2187	0.5033	10.37	<0.0001
**Annual**	Cycle 1^e^	Stochastic disturbance (*σ*^2^_*υ*_)	0.0389	0.04305	0.90	0.3667	9212.4917	5069.2	1.82	0.0692
**Annual**	Cycle 2^f^	Damping factor (*ρ*)	1.0000	0.0003099	3227.06	<0.0001	0.5300	0.3188	1.66	0.0964
**Annual**	Cycle 2^f^	Period (*p*)	2.1991	0.01326	165.88	<0.0001	2.3371	0.2958	7.90	<0.0001
**Annual**	Cycle 2^f^	Stochastic disturbance (*σ*^2^_*υ*_)	0.0437	0.04930	0.89	0.3754	19117	8719.6	2.19	0.0283
**Wet**	Irregular^d^	Stochastic disturbance (*σ*^2^_*ϵ*_)	14683	3448.3	4.26	<0.0001	38246	6563.8	5.83	<0.0001
**Wet**	Level	Stochastic disturbance (*σ*^2^_*η*_)	0.00000114	0.01930	0.00	1.0000	105.1059	270.5242	0.39	0.6976
**Wet**	Cycle 1^e^	Damping factor (*ρ*)	0.9939	0.03057	32.52	<0.0001	0.9747	0.04195	23.23	<0.0001
**Wet**	Cycle 1^e^	Period (*p*)	2.9893	0.04784	62.48	<0.0001	5.2113	0.1585	32.89	<0.0001
**Wet**	Cycle 1^e^	Stochastic disturbance (*σ*^2^_*υ*_)	41.7160	47.80989	0.87	0.3829	231.4622	217.5619	1.06	0.2874
Wet	Cycle 2^f^	Damping factor (*ρ*)	1.0000	0.0002705	3696.71	<0.0001	1.0000	0.0001453	6881.19	<0.0001
Wet	Cycle 2^f^	Period (*p*)	2.19466	0.01747	125.60	<0.0001	2.5281	0.01315	192.29	<0.0001
Wet	Cycle 2^f^	Stochastic disturbance (*σ*^2^_*υ*_)	0.02091	0.02545	0.82	0.4114	0.0154	0.01932	0.80	0.4263
Dry	Irregular^d^	Stochastic disturbance (*σ*^2^_*ϵ*_)	5629.9394	1161.4	4.85	<0.0001	2877.0765	526.6863	5.46	<0.0001
Dry	Level	Stochastic disturbance (*σ*^2^_*η*_)	3.8476E‒7	0.0072965	0.00	1.0000	20.4084	59.3828	0.34	0.7311
Dry	Cycle	Damping factor (*ρ*)	1.0000	0.0002902	3445.35	<0.0001	1.0000	0.0001631	6129.68	<0.0001
Dry	Cycle	Period (*p*)	3.0271	0.04221	71.72	<0.0001	2.2526	0.0092495	243.53	<0.0001
Dry	Cycle	Stochastic disturbance (*σ*^2^_*υ*_)	0.0031	0.0045064	0.68	0.4969	0.00117	0.001427	0.82	0.4134

^a^Rainfall recordings were derived from 15 gauges (Eq 3 in S1 Text) during 1966–2014 (dry season: 1965–2014).

^b^Rainfall was recorded during 1914–2015 (dry season: 1913–2015).

^c^Based on a t-test.

^d^Overall residual.

^e^Primary cycle.

^f^Secondary cycle. Significant stochastic cycle disturbances are marked in bold-faced font.

The stochastic cycle disturbance variances were insignificant except for the secondary 2.3-year annual rainfall cycle in Narok Town (Table 2). The stochastic disturbance variance of the primary annual cycle did not quite reach significance (Table 2). The 3-year cycles for the primary annual and wet season rainfall and the secondary 2.2-year cycles for the annual and wet season were significant for the Mara in the final state model at the end of the estimation span (Table 3). For Narok Town, the cyclic rainfall components were significant for the 2.5-year secondary wet season rainfall cycle and for the 2.3-year dry season rainfall cycle (Table 3).

**Table 3:** Significance analysis of components (based on the final state) of annual, wet and dry season rainfall components.

		Mara^a^			Narok^b^		
Rainfall component	Model component	Degrees of freedom	Chi-Square	Approx. P-value^c^	Degrees of freedom	Chi-Square	Approx. P-value^c^
**Annual**	Irregular (*ϵ*_*t*_)^d^	1	6.43	0.0112	1	0.00	0.9994
**Annual**	Level (*μ*_*t*_)	1	1919.26	<0.0001	1	142.10	<0.0001
**Annual**	Cycle 1 (*φ*_*t*_)^e^	2	18.72	<0.0001	2	0.02	0.9897
**Annual**	Cycle 2 (*φ*_*t*_)^f^	2	15.80	0.0004	2	0.46	0.7962
**Wet**	Irregular (*ϵ*_*t*_)^d^	1	0.91	0.3400	1	0.03	0.8667
**Wet**	Level (*μ*_*t*_)	1	1986.76	<0.0001	1	189.89	<0.0001
**Wet**	Cycle 1 (*φ*_*t*_)^e^	2	6.99	0.0304	2	0.32	0.8534
**Wet**	Cycle 2 (*φ*_*t*_)^f^	2	9.42	0.0090	2	7.80	0.0202
**Dry**	Irregular (*ϵ*_*t*_)^d^	1	119.55	<0.0001	1	6.20	0.0128
**Dry**	Level (*μ*_*t*_)	1	397.42	<0.0001	1	79.22	<0.0001
**Dry**	Cycle 1 (*φ*_*t*_)	2	4.27	0.1181	2	9.08	0.0107

^a^Rainfall recordings were derived from 15 gauges (Eq 3 in S1 Text) during 1966–2014 (dry season: 1965–2014).

^b^Rainfall was recorded during 1914–2015 (dry season: 1913–2015).

^c^Based on a Wald test.

^d^Overall residual.

^e^Primary cycle.

^f^Secondary cycle. Significant components are marked in bold-faced font.

The damping factors for the rainfall cycles were approximately 1 except for the primary (0.76) and secondary (0.53) annual rainfall cycles in Narok Town (Table 2). These properties collectively imply that the annual and wet season rainfall cycles were deterministic and persistent in the Mara. For Narok Town, the primary dry season cycle and the secondary wet season cycle were also non-random and persistent. The annual rainfall cycles and the primary wet season cycle in Narok Town contained stochastic and transient elements.

### Changing periodicity in monthly rainfall oscillations

**Fig 4:**
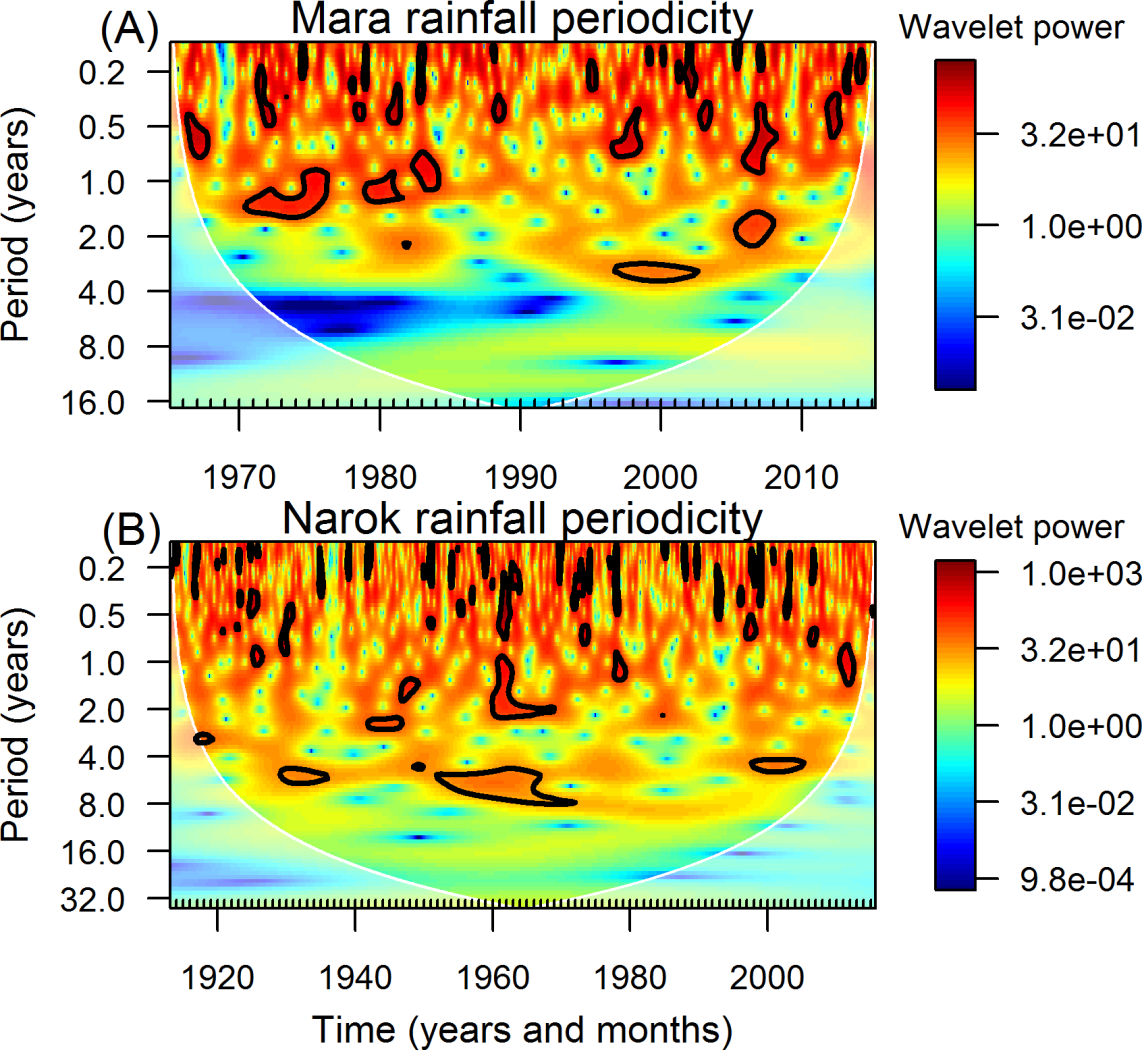
Periodicity in monthly rainfall derived from wavelet analysis. (A) Rainfall recordings in the Mara were derived from 15 gauges (Eq 3 in S1 Text) during the period 1965–2015. (B) Rainfall in Narok Town in Kenya was recorded during 1913–2015. The wavelet power spectrum (bias-corrected and normalized by the variance) for varying rainfall cycle periods is given for each month and year. Areas of high power are indicated in warm colours (red), whereas areas of low power are indicated in cold colours (blue). The semi-transparent area represents the cone of influence, where edge effects become important due to padding with zeros at the end of the time series. The power in this region is reduced. Significantly higher wavelet power spectra than expected under a red-noise process AR(1) (lag ‒ 1 = 0.05 for the Mara and lag ‒ 1 = 0.16 for Narok Town) are encircled by black lines.

Wavelet analysis established the existence of oscillations in rainfall with significantly time-varying cycle periods (periodicity). The statistically significant cycles identified for rainfall in the Mara by wavelet analysis ranged from about 0.75 to 1.5, 1.5 to 2.5 and 2.5 to 3.5 years (Fig 4A). For Narok Town, statistically significant cycles of about 0.75 to 1.5, 1 to 2.5, 2 to 3, 4 to 6 and 4.5 to 8 years were identified (Fig 4B). Seasonal rainfall oscillations with significant cycles of less than one year were distributed throughout the full span of the time series as expected (Fig 4).

During 1970–1984 oscillations with a periodicity of about 0.75 to 1.5 years were apparent during three distinct episodes in the Mara: 1970–1976, 1979–1981 and 1982–1984 (Fig 3A). Significant but longer 2.5 to 3.5-year cycles characterised the period around the turn of the second millennium (1996–2002; Fig 4A). Furthermore, significant rainfall cycles of about 1.5 to 2.6 years were evident in the Mara during 2005–2007 (Fig 4A).

For Narok Town, significant 2 to 3-year rainfall cycles were apparent during the periods 1916–1919 and 1941–1946. The latter period was followed by significant oscillations of about 1.5 to 2 years during 1946–1949 (Fig 4B). Significant but longer 4 to 6-year cycles occurred during 1929–1935 and during 1997–2005 (Fig 4B). Additionally, long and significant 4.5 to 8-year cycles characterized the period 1951–1971 (Fig 4B). The episode partially overlapped with a significant, about 1 to 2.5-year cycle during 1959–1968 (Fig 4B). An oscillation with a short, approximately 0.75 to 1.5-year cycle was apparent during 2010–2012 and another during 1977–1979 (Fig 4B).

### Frequency, severity and timing of droughts and floods

**Fig 5:**
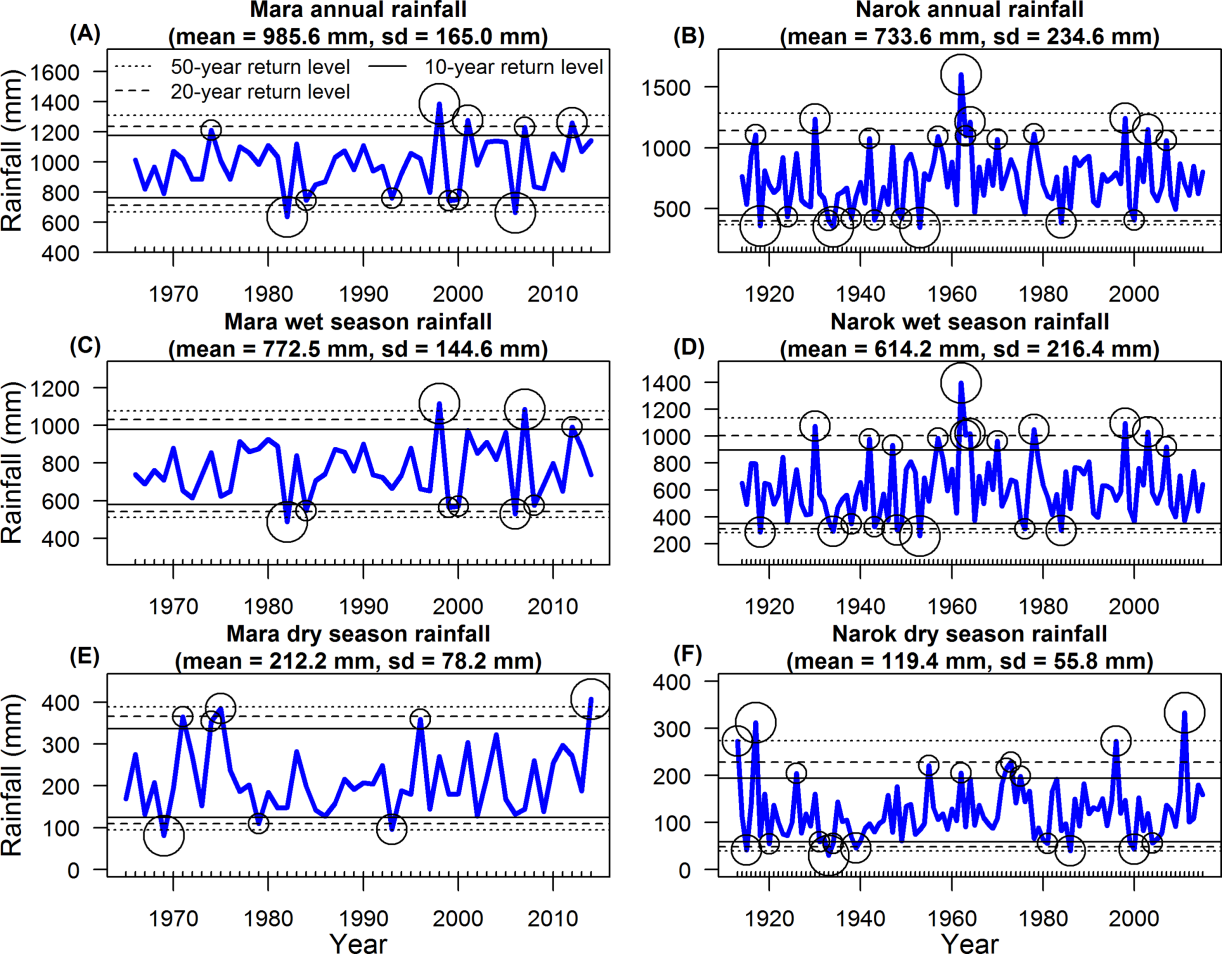
Extreme values (circles) for the annual, wet season and dry season rainfall components. The thin lines are the 50-year (dotted), 20-year (dashed) and 10-year (continuous) return levels. (A, C, E) Rainfall recordings in the Mara were derived from 15 gauges (Eq 3 in S1 Text) during 1966–2014 (dry season: 1965–2014). (B, D, F) Rainfall in Narok Town in Kenya was recorded during 1914–2015 (dry season: 1913–2015). The (A, B) annual, (C, D) wet season and (E, F) dry season rainfall components (thick blue lines) were summed from the monthly rainfall records.

The temporal sequences of all severe droughts (below the 10-year return level) and floods (above the 10-year return level) of the annual and seasonal rainfall components for the Mara during 1966–2014 (dry season: 1965–2014) and for Narok Town during 1914–2015 (dry season: 1913–2015) are given in Fig 5. The return levels of extreme annual, wet season and dry season rainfall components for return periods up to 50 years for the Mara and Narok Town estimated using extreme value analysis techniques are shown in S7 Fig.

The number of severe annual (Fig 5A) and wet season (Fig 5C) droughts in the Mara rainfall time series doubled from 2 during 1965–1989 to 4 during 1990–2014. The worst annual and wet season drought did not reach the 50-year return level in 1982 (Fig 5A), followed by 2006, in which rainfall was below the 20-year return level (Fig 5C). The extreme drought of 2006 was coincident with rainfall oscillations with significant 1.5 and 2.5-year cycles (Fig 4A).

All the annual and wet season floods occurred during 1998–2012 in the Mara except for one severe annual flood in 1974 (Fig 5A and 5C). Of those, the flood in 1998 was the most extreme, exceeding the 50-year return levels (Fig 5A and 5C). This flood occurred during a time of significant rainfall periodicity of 2.5 to 3.5 years in the Mara (Fig 4A) and 4 to 6 years in Narok Town (Fig 4B).

Severe dry season droughts or floods did not increase in frequency (Fig 5E). The worst dry season drought was in 1969 with rainfall falling below the 50-year return level (Fig 5E). Another extreme drought, in which rainfall did not reach the 20-year return level, occurred during 1993 (Fig 5E). The highest dry season rainfall exceeded the 50-year return level in 2014 in the Mara (Fig 5E).

In contrast to the Mara, the frequency of severe droughts or floods did not increase in Narok Town (Fig 5B, 5D and 5F). Annual (Fig 5B), wet season (Fig 5D) and dry season (Fig 5F) droughts were more frequent up to 1953, the year with the lowest annual and wet season rainfall. The most severe drought during the dry season occurred in 1933 in Narok (Fig 5F). The droughts of 1933 and 1953 were contemporaneous with rainfall oscillations with significant, approximately 6-year cycles (Fig 4B). The majority of the severe annual (Fig 5B) and wet season (Fig 5D) floods were distributed around the most extreme flood year in 1962, in which rainfall was well above the 50-year return levels in Narok. This flood was associated with rainfall oscillations with long and significant 4.5 to 8-year cycles lasting from 1951 to 1971 (Fig 4B). The most extreme dry season rainfall exceeded the 50-year return level in 1912 and in 2011 (Fig 5F).

These results demonstrate that the severe annual and wet season droughts and floods became more frequent during 1991–2014 than during the earlier years (1965–1990) in the Mara. The severe annual and wet season floods in Narok Town were clustered around the most extreme flood year of 1962.

### Multiannual persistence of severe droughts and floods

The extremal runs index established multiannual persistence in the sequence of severe droughts (below the 10-year return level) and floods (above the 10-year return level) except for the dry season droughts in the Mara, the wet season droughts in Narok Town and the annual and wet season floods in the Mara (Table 4). The extremal interval index established clustering in the sequence of severe wet season droughts in the Mara, and in the sequence of severe dry season droughts in Narok Town (Table 4). The confidence intervals for the extremal interval index for the severe dry season droughts in Narok Town did not include unity (value 1), indicating significant clustering (Table 4). All the other confidence intervals for the estimated extremal indices included unity (indicating no clustering; Table 4). The runs index and its confidence limits are unity if there is no single instance of multiannual persistence of severe drought or flood years in the annual or seasonal rainfall components. The interval index and its confidence limits are unity if the time series contains only 3 or fewer severe drought and flood years separated by 2 or less inter-exceedance times as apparent from Eq 2 in S5 Text.

**Table 4:** Extremal runs and interval indices and significance of runs tests for clustering of severe drought and flood years.

Extreme events	Test statistics	Annual rainfall		Wet season rainfall		Dry season rainfall	
		Mara^a^	Narok^b^	Mara^a^	Narok^b^	Mara^a^	Narok^b^
**Severe** **droughts**^**c**^	Extremal runs index (95% CI)^g^	**0.83 (0.63–1.00)**	**0.90 (0.75–1.00)**	**0.83 (0.63–1.00)**	1.00 (1.00–1.00)	1.00 (1.00–1.00)	**0.90 (0.75–1.00)**
	Extremal interval index (95% CI)^h^	1.00 (0.99–1.00)	**0.99 (0.74–1.00)**	**0.87 (0.53–1.00)**	1.00 (1.00–1.00)	1.00 (1.00–1.00)	**0.74 (0.52–0.74)**
	Runs test	0.503	0.625	0.503	1.000	1.000	0.621
**Extreme droughts**^**d**^	Extremal runs index (95% CI)^g^	1.00 (1.00–1.00)	1.00 (1.00–1.00)	1.00 (1.00–1.00)	1.00 (1.00–1.00)	1.00 (1.00–1.00)	1.00 (1.00–1.00)
	Extremal interval index (95% CI)^h^	1.00 (1.00–1.00)	1.00 (1.00–1.00)	1.00 (1.00–1.00)	1.00 (1.00–1.00)	1.00 (1.00–1.00)	1.00 (1.00–1.00)
	Runs test	1.000	1.000	1.000	1.000	1.000	1.000
**Severe floods**^**e**^	Extremal runs index (95% CI)^g^	1.00 (1.00–1.00)	**0.83 (0.63–0.83)**	1.00 (1.00–1.00)	**0.83 (0.63–1.00)**	**0.80 (0.57–1.00)**	**0.90 (0.75–1.00)**
	Extremal interval index (95% CI)^h^	1.00 (0.92–1.00)	1.00 (0.90–1.00)	1.00 (1.00–1.00)	1.00 (0.86–1.00)	1.00 (0.64–1.00)	1.00 (0.82–1.00)
	Runs test	1.000	0.386	1.000	0.386	0.097	0.285
**Extreme floods**^**f**^	Extremal runs index (95% CI)^g^	1.00 (1.00–1.00)	1.00 (1.00–1.00)	1.00 (1.00–1.00)	**0.71 (0.45–1.00)**	1.00 (1.00–1.00)	1.00 (1.00–1.00)
	Extremal interval index (95% CI)^h^	1.00 (1.00–1.00)	1.00 (0.68–1.00)	1.00 (1.00–1.00)	1.00 (0.60–1.00)	1.00 (1.00–1.00)	**0.97 (0.77–1.00)**
	Runs test	1.000	1.000	1.000	**0.054**^**i**^	0.118	0.183

^a^Rainfall recordings were derived from 15 gauges (Eq 3 in S1 Text) during 1966–2014 (dry season: 1965–2014).

^b^Rainfall was recorded during 1914–2015 (dry season: 1913–2015). Monthly rainfall records were summed to calculate the annual and seasonal rainfall components.

^c^Below the 10- return levels.

^d^Below the 20-year return levels.

^e^Above the 10-year return levels.

^f^Above the 20-year return levels.

^g^Index based on run length of 1 year and deviations from unity are indicated in bold-faced font.

^h^Deviations from unity are indicated in bold-faced font.

^i^P-value indicates marginal significance.

The p-value for the runs test was marginally significant (Table 4), suggesting that the sequence of extreme flood years in which the wet season rainfall exceeded the 20-year return level in Narok Town deviated from complete randomness. Otherwise, runs tests did not establish any other significant deviation from randomness in the occurrence of severe droughts and floods in the time series of rainfall for the Mara or Narok Town (Table 4).

### Trends in the severity of droughts and floods

**Fig 6:**
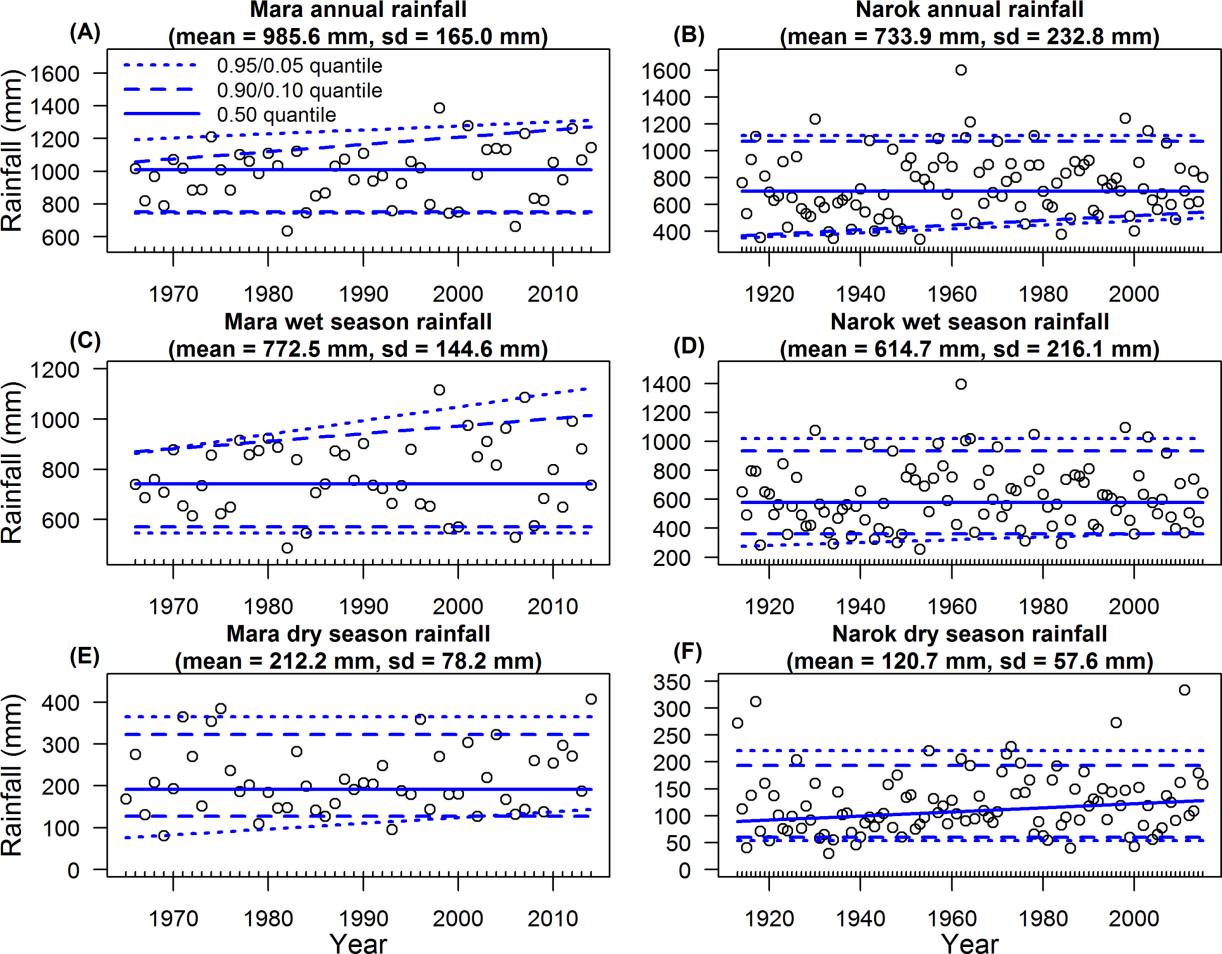
Temporal trends in the severity of droughts and floods. Blue lines are the 0.95/0.05 (dotted), 0.90/0.10 (dashed) and 0.50 (continuous) quantiles. (A, C, E) Rainfall recordings in the Mara were derived from 15 gauges (Eq 3 in S1 Text) during 1966–2014 (dry season: 1965–2014). (B, D, F) Rainfall in Narok Town in Kenya was recorded during 1914–2015 (dry season: 1913–2015). The (A, B) annual, (C, D) wet season and (E, F) dry season rainfall components (points) were summed from the monthly rainfall records.

There was no evidence for a significant trend in the severity of droughts in the Mara from quantile regression during 1965–2014 (Table 5, Fig 6A, 6C and 6E). In Narok Town, the 0.10 quantile for the annual rainfall component increased significantly during 1914–2015 (Table 5, Fig 6B), indicating decreasing severity of droughts. The apparent decrease in the severity of the extreme (0.05 quantile) dry season droughts in the Mara (Fig 6E) and the decrease in extreme annual (Fig 6B) and wet season (Fig 6D) droughts in Narok Town were not significant (Table 5).

**Table 5:** Temporal trends in rainfall extremes (0.05, 0.10, 0.50, 0.90 and 0.95 quantiles).

		Mara^a^						Narok^b^					
Rainfall component	Quantile	ΔAICc^c^	Effects	Estimate	Std. Error	t Value	Pr. > |t|	ΔAICc^c^	Effects	Estimate	Std. Error	t Value	Pr. > |t|
**Annual rainfall**	**0.05**	0.0	Intercept	744	47	15.9	<0.0001	4.2	Intercept	425	28	15.1	<0.0001
									Year	440	251	1.6	0.083
	**0.10**	0.0	Intercept	752	34	22.3	<0.0001	8.7	Intercept	454	22	21.0	<0.0001
									Year	526	243	2.2	0.0326
	**0.50**	0.0	Intercept	1008	31	32.6	<0.0001	0.0	Intercept	698	30	22.3	<0.0001
	**0.90**	10.8	Intercept	1162	40	29.0	<0.0001	0.0	Intercept	1071	61	17.6	<0.0001
			Year	452	239	1.9	0.0648						
	**0.95**	4.4	Intercept	1251	61	20.5	<0.0001	0.0	Intercept	1115	55	20.3	<0.0001
			Year	249	323	0.8	0.4448						
**Wet season rainfall**	**0.05**	0.0	Intercept	546	29	19.0	<0.0001	4.8	Intercept	324	20	16.2	<0.0001
									Year	296	170	1.7	0.0856
	**0.10**	0.0	Intercept	741	32	17.6	<0.0001	0.0	Intercept	362	19	19.2	<0.0001
	**0.50**	0.0	Intercept	742	39	19.1	<0.0001	0.0	Intercept	578	27	21.1	<0.0001
	**0.90**	10.5	Intercept	941	32	29.9	<0.0001	0.0	Intercept	952	65	14.7	<0.0001
			Year	306	222	1.4	0.1742						
	**0.95**	17.9	Intercept	991	41	24.1	<0.0001	0.0	Intercept	1021	43	23.7	<0.0001
			**Year**	556	268	2.1	0.0413						
**Dry season rainfall**	**0.05**	5.9	Intercept	110	12	9.4	<0.0001	0.0	Intercept	54	6	8.9	<0.0001
			Year	141	86	1.6	0.108						
	**0.10**	0.0	Intercept	127	11	11.2	<0.0001	0.0	Intercept	60	4	14.2	<0.0001
	**0.50**	0.0	Intercept	191	10	18.9	<0.0001	3.3	Intercept	109	6	17.9	<0.0001
									Year	115	70	1.6	0.1048
	**0.90**	0.0	Intercept	322	32	10.1	<0.0001	0.0	Intercept	193	14	13.9	<0.0001
	**0.95**	0.0	Intercept	366	24	15.2	<0.0001	0.0	Intercept	221	27	8.1	<0.0001

^a^Rainfall recordings were derived from 15 gauges (Eq 3 in S1 Text) during 1966–2014 (dry season: 1965–2014).

^b^Rainfall was recorded during 1914–2015 (dry season: 1913–2015). The monthly rainfall records were summed to yield the annual and seasonal rainfall components.

^c^ΔAICc are the deviations in AICc values of each model from that for the null (intercept-only) model for each quantile. Significant trends in rainfall quantiles are marked in bold-faced font.

Extreme wet season floods (0.95 quantile) increased significantly in the Mara during 1966–2014 (Table 5, Fig 6C). The increase in severe annual floods (0.90 quantile) was close to significance in the Mara (Table 5, Fig 6A). The increase in the extreme (0.95 quantile) annual rainfall component (Fig 6A) and increase in the 0.90 quantile of the wet season rainfall component (Fig 6C) in the Mara were not significant (Table 5).

The median (0.50 quantile) dry season rainfall component increased substantially though non-significantly from about 90 mm in 1913 to about 125 mm in 2015 in Narok Town (Table 5, Fig 6F). Otherwise, we found no evidence for significant trend over time in the median annual or seasonal rainfall components for the Mara or Narok Town (Table 5, Fig 6).

## Discussion

We analysed temporal trends and variation in rainfall in the Maasai Mara ecosystem in Kenya as a background for understanding animal population and biodiversity dynamics in African terrestrial ecosystems. In contrast to IPCC’s predictions [2] and projections of the General Circulation Models [33–35], which have difficulties capturing small-scale orographic rainfall variation [96], we found only minor empirical support for increases in rainfall over two areas in Eastern Africa.

Our results show that only the dry season rainfall component for Narok Town increased during 1940–2015 based on UCM models. Likewise, the dry season rainfall increased during 1913–2001 in the Serengeti [52]. But our finding of decreasing annual rainfall during 1962–2015 in Narok Town reinforces results of earlier studies [36,44–46] that attributed decreasing East African rainfall to increasing temperatures of the northern hemisphere [36] and of the central Indian or west Pacific Oceans [44–46]. Similarly, Ritchie et al. [52] documented evidence of decreasing annual and wet season rainfall during 1960–2001 in the Serengeti. Rainfall also declined during 1960–2014 in 14 Kenyan counties but in another 6 Kenyan counties it declined initially and then switched to an upward trend according to Ogutu et al. [30], demonstrating spatial distinctions in rainfall trends. There was, however, no systematic change in the annual and seasonal rainfall components during 1965–2014 in the Mara according to our UCM analysis. The apparent discrepancies in the contrasting findings may partly reflect the strong spatial variation evident in East-African rainfall [55,97] and calls for considerable caution in applying results of climate modelling at large spatial scales to particular localities.

Our result demonstrating virtually constant rainfall seasonality in the Mara or Narok Town suggests that regular movements of the Inter-Tropical Convergence Zone provide an extremely stable modulation of the rainfall seasons. The higher and less variable rainfall in the Mara than in Narok Town, in particular the higher and less variable dry season rainfall, is likely due to the closer proximity of the Mara to the high-precipitation areas near the eastern shores of Lake Victoria [55] and the influence of the Lake Victoria System. The modulation of Mara’s rainfall by this system and the resulting higher dry season rainfall likely sustains the high abundance and diversity of wildlife and livestock there. The seasonal variation in rainfall both in the Mara and Narok Town agree well with the monthly discharge patterns for the Mara River, with peaks in December and April-May [98], implying that rainfall governs much of the Mara River discharge.

The compensatory pattern we detected for the amounts of the wet and dry season rainfall, where periods with below-average dry season rainfall frequently received above-average wet season rainfall and vice versa, may possibly emerge from the interactions between the local Lake Victoria circulation and hemispheric level climate drivers. This compensatory dampening of rainfall seasonality has likely generated the stability and resilience of the Mara and allowed the high abundance and diversity of wildlife it supports. The positive Indian Ocean Dipole Mode and the El Niño–Southern Oscillation can increase East African rainfall primarily during the wet season by weakening westerly winds and enabling moisture transport from the Indian Ocean towards the land [42,43,99]. But weakened westerly winds may, in turn, reduce the dry season rainfall that typically originates from Lake Victoria and precipitates east of the lake in the Mara [55]. Correspondingly, La Niña-like conditions and negative Indian Ocean Dipole phases may enhance westerly winds from Lake Victoria that lead to above-average dry season rainfall but concurrently block moisture export from the Indian Ocean towards East Africa resulting in less wet season rainfall.

The predominantly persistent rainfall cycle periods we estimated of between 2.1 and 3 years for the Mara and 2.3 to 5.2 years for Narok Town agree with the dominant cycle periods reported for East Africa ranging from about 2 to 11 years [56–58,76]. They are also within the range of the periods characteristic of the Indian Ocean Dipole (about 2 years), the El Niño-Southern Oscillation (3–6 years; [100]) and the quasi-biennial oscillations in the lower equatorial stratospheric zonal winds [101].

By means of wavelet analysis we were able to establish that periodicity in the monthly rainfall oscillations is not constant but varies over time and space in the Mara-Serengeti ecosystem. The cycle periods varied from just 0.75 to 1.5 years for several distinct episodes during 1970–1984 for the Mara to periods ranging from 4.5 to 8 years during 1951–1971 for Narok Town. Likewise, the amplitude and frequency of the Indian Ocean Dipole and the El Niño-Southern Oscillation can be highly variable [100,102].

In general, the wavelet analysis showed that extreme droughts and floods coincided with the times when the regional rainfall oscillations had statistically significant cycle periods. These results strongly suggest a possible association between oscillations in hemispheric atmospheric and oceanic circulations and regional rainfall oscillations in the Mara-Serengeti ecosystem that are similarly evident in East African rainfall [103–105]. For example, the first half of the wet season of 2006 was associated with both a strong negative Indian Ocean Dipole and La Niña-like conditions [50,51] with consequent severe drying in East Africa [48,75,82]. The flood of 1962 coincided with strong easterly winds [106] and an extreme dipole reversal of the Indian Ocean sea surface temperatures [42]. But there was no simultaneous El Niño effect during that time [42]. Similarly, the flood of 1998 was associated with the second strongest El Niño event on instrumental record during 1950–2016 [50], and coincided with a strong and positive Indian Ocean Dipole [42,50,51]. These circulations may also explain the simultaneous occurrence of overlapping rainfall cycles that were apparent both in UCM models and wavelet analysis.

But the extreme dry season droughts of 1969 and 1993, and the extreme annual and wet season drought of 1982 in the Mara were not associated with any significant periodicity in rainfall oscillation. There was a moderate El Niño event during 1968–1969 and 1993, and the longest recorded El Niño episode during 1990–1995 [50]. The interactions between the El Niño events and Lake Victoria circulation patterns that probably underlie the compensatory pattern of the wet and dry season rainfall could also have contributed to the dry season droughts of 1969 and 1993.

Our results indicating increasing frequency and severity of floods in the Mara during 1965–2014 and reduction in drought frequency and severity in Narok Town during 1913–2015 agree with the projections of General Circulation Models for most of East Africa of more intense wet seasons [107] and less severe droughts [74]. Precipitation extremes are expected as temperatures rise [107]. Such patterns may emerge from the projected strengthening of the El Niño Southern Oscillation [108,109] and more frequent occurrences of the positive phases of the Indian Ocean Dipole [38,39].

In contrast to Narok, severe droughts are apparently becoming more frequent in the Mara. Although some of the inconsistencies in our results might have arisen from using only one gauge in Narok Town versus 15 in the Mara, they show that the trends in extreme rainfall events can vary both locally and over time. Simulation models indicate that, although droughts may increase with increasing temperatures on a global scale, the changes expected for East Africa appear uncertain and comparatively small [47]. One possible cause of the recent severe droughts in the Mara could be due to the increasing frequency of extreme La Niña events that often follow extreme El Niño events [47]. These hemispheric circulation patterns may also underlie the multiannual persistence of severe droughts and floods that we detected in the annual and seasonal rainfall components in the Mara and in Narok Town.

### Implications for animal population and biodiversity dynamics

The decreasing annual rainfall in Narok Town may have negative long-term effects on the reproductive performance of herbivores, including livestock, in that area through limitation of food and surface water availability [5,6,10,110]. Wildlife and livestock populations are also likely to suffer more from water and food scarcity in the Mara despite the stable rainfall levels during 1965–2014 due to the rising temperatures and increasing human impacts [30]. In addition, the combination of decreasing rainfall, rising temperatures and hence increased rates of evapo-transpiration can make wildfires more destructive and severe [24]. Such conditions can have potentially adverse effects on animal populations and biodiversity [30]. By contrast, the increase in dry season rainfall in Narok Town during 1940–2015 can improve the survival prospects of ungulates when resources are most limiting [10,16].

The predominantly deterministic and persistent primary rainfall cycles in the Mara likely have important implications for the dynamics and management of animal populations and their vegetation resources [5,14]. The changing periodicity in the rainfall oscillations and the increasing amplitude in the wet season rainfall oscillations in the Mara may compound impacts of the recurrent seasonal and cyclic variations in water and forage availability that animals already have to cope with by adaptively adjusting their reproduction, foraging and migration patterns.

The increase in excessive wet season rainfall in the Mara can displace wildlife due to flooding [29,111], reduce recruitment [112] and forage quality due to excessive plant growth and dilution of plant nutrients and cause population declines [28,74]. The increasing severity of floods creates favourable conditions for the transmission of several diseases, including anthrax [113], Rift Valley Fever [114] and African horse sickness [115] and promotes infestation with parasites [20]. In the Serengeti, abnormally high wet season rainfall may enable wildebeest to use the nutrient-rich southern short grass plains for extended periods towards the dry season [25]. As a consequence, the excessive rainfall may reduce wildebeest’s occupancy of their dry season range in the Mara in Kenya [25]. Rainfall-mediated migrations and local concentrations of large herbivores can have cascading and difficult-to-predict effects on the ecosystem by modifying nutrient cycling through grazing, urine and dung deposition [116] and through prey availability for predators and scavengers [117,118].

If the pattern of increasing drought frequency continues in the Mara it will likely have adverse and immediate impacts on both wildlife and livestock populations in the ecosystem [16,29,75,80,82,119], although some uncertainty remains owing to the effect of the opposing trend in drought frequency in Narok Town. Droughts also tend to be associated with outbreaks of infectious diseases among large herbivores [120,121], such as anthrax that spreads when herbivores graze short grass close to the ground. In particular, multi-year droughts and floods can have much stronger adverse impacts on herbivores than single-year droughts [15,75]. The changing drought and flood frequencies can also affect biodiversity in the region through their impact on the Mara River flow levels.

## Conclusions

For important and unique ecosystems such as the Maasai Mara in East Africa it is crucial to keep reliable historical and comprehensive contemporary climate records to provide a sound scientific basis for informing decisions on the nature and consequences of climate change to large herbivores and their predators, biodiversity and human livelihoods. Our analysis of verified station data for the Mara region and Narok Town in Kenya indicates that local trends and variation in rainfall can differ substantially from the patterns predicted for regional or continental scales. In particular, we found only minor support for IPCC’s large-scale predictions of very likely increases in East African rainfall. Although the rainfall cycle periods in our study area correspond to the dominant cycle periods evident in East African rainfall, we found strong spatial and temporal variation in rainfall periodicity in the Maasai Mara ecosystem. While droughts are apparently becoming more frequent in the Mara, we detected the opposite pattern for Narok Town located only 75 km away. Similarly, wet season floods became more severe in the Mara but not in Narok Town. Significant changes in rainfall amounts and periodicity can profoundly affect animal population and biodiversity dynamics by lowering the availability and increasing the variability of food and water resources, increasing the risk of outbreaks of infectious diseases and altering ungulate migration and dispersal patterns.

## Supporting information

S1 Data Mara monthly rainfall 1965–2015. Rainfall data comprising recordings from 15 stations in the Mara region of Kenya. KMD, Kenya Meteorological Department; MMEMP, Maasai Mara Ecological Monitoring Programme; NA, not available; Rainfall (mm), records verified for analysis; Source of verification; Rain_imp, rainfall records in mm including imputed values used in analyses.
(XLSX)

S2 Data Narok monthly rainfall 1913–2015. Rainfall data comprising recordings from the gauge in Narok Town operated by the Kenya Meteorological Department.
(XLSX)

S1 Text Rainfall standardisation methods. (DOCX)

S2 Text Modelling seasonal oscillations in rainfall. (DOCX)

S3 Text Using wavelet analysis to detect changing periodicity in monthly rainfall oscillations. (DOCX)

S4 Text Threshold selection for estimating return levels of droughts and floods. (DOCX)

S5 Text Estimation of the runs and intervals estimator to detect multiannual persistence of droughts and floods. (DOCX)

S6 Text Quantile regression to analyse trends in the severity of droughts and floods. (DOCX)

S1 File R code used to analyse the time series of rainfall derived from 15 gauges in the Mara (1966–2014) and a gauge in Narok Town (1913–2015). The code is provided for wavelet analysis for the total monthly rainfall, and extreme value analysis, extremal indices, runs tests and quantile regression for the annual and seasonal rainfall components. The standardisation methods for the Mara data are included.
(TXT)

S2 File SAS code used to fit UCM models for the time series of rainfall derived from 15 gauges in the Mara (1966–2014) and a gauge in Narok Town (1913–2015). The analysis pertains to the time series of the total monthly rainfall, and to the annual and seasonal rainfall components.
(TXT)

S1 Fig Standardisation methods for the time series of annual rainfall derived from 15 gauges in the Mara (1966–2014). Four different standardisation methods were applied: arithmetic mean (black line), mean-weighted average (orange line), gauge-adjusted mean (blue line) and Best Linear Unbiased Predictions (BLUPS) from a generalized linear mixed model with Tweedie error distribution and a log link function (red line).
(TIFF)

S2 Fig Smoothed level component based on the structural time series analysis of monthly rainfall. (A) Rainfall recordings in the Mara were derived from 15 gauges (Eq 3 in S1 Text) recording in the Mara during 1965–2014 and in (B) Narok Town in Kenya during 1913–2015. Significant components are marked in bold-faced font.
(TIF)

S3 Fig Cumulative plot of decadal averages of the total monthly rainfall. (A) Rainfall in the Mara during 1965–2014. (B) Rainfall in Narok Town in Kenya during 1913–2015.
(TIF)

S4 Fig The standardized (divided by the mean) moving averages of the wet season (red lines) and dry season (blue lines) rainfall components. The vertical needles are the standardized deviates and the solid curves are the 3-year (Mara), 5-year (wet season of Narok Town) and 2-year (dry season of Narok Town) moving averages. (A) Rainfall recordings in the Mara were derived from 15 gauges (Eq 3 in S1 Text) during 1966–2014 (dry season: 1965–2014). (B) Rainfall in Narok Town in Kenya was recorded during 1914–2015 (dry season: 1913–2015). The wet season and dry season rainfall components were summed from the monthly rainfall records.
(TIF)

S5 Fig Smoothed primary cycles for standardized rainfall based on the structural time series analysis. (A, C, D) Rainfall recordings in the Mara were derived from 15 gauges (Eq 3 in S1 Text) during 1966–2014 (dry season: 1965–2014). (B, D, F) Rainfall in Narok Town in Kenya was recorded during 1914–2015 (dry season: 1913–2015). The (A, B) annual, (C, D) wet season and (E, F) dry season rainfall components were summed from the monthly rainfall records.
(TIF)

S6 Fig Smoothed secondary cycles for standardized rainfall based on the structural time series analysis. (A, C) Rainfall recordings in the Mara were derived from 15 gauges (Eq 3 in S1 Text) during 1966–2014 (dry season: 1965–2014). (B, D) Rainfall in Narok Town in Kenya was recorded during 1914–2015 (dry season: 1913–2015). The (A, B) annual and (C, D) wet season rainfall components were summed from the monthly rainfall records.
(TIF)

S7 Fig Return periods and the corresponding return levels (blue lines) for extreme rainfall. (A, B, E, F, I, J) Rainfall recordings in the Mara were derived from 15 gauges (Eq 3 in S1 Text) during 1966–2014 (dry season: 1965–2014). (C, D, G, H, K, L) Rainfall in Narok Town in Kenya was recorded during 1914–2015 (dry season: 1913–2015). The (A, B, C, D) annual, (E, F, G, H) wet season and (I, J, K, L) dry season rainfall components were summed from the monthly rainfall records. Blue polygons are the 95% normal approximate confidence bands.
(TIFF)

S1 Table Significance analysis of components (based on the final state) of monthly rainfall. (XLSX)

S2 Table The estimated variances of the disturbance terms and the damping factor of the autoregressive component for the monthly rainfall series. (XLSX)
