# Supplementary material for: Rainfall trends and variation in the Maasai Mara ecosystem and their implications for animal population and biodiversity dynamics
Source: PLoS One. 2018 Sep 19;13(9):e0202814. doi: 10.1371/journal.pone.0202814 (PMC6145597; doi:10.1371/journal.pone.0202814)
Supplement: S1 Text — (DOCX) [file pone.0202814.s003.docx]

# S1 Text. Rainfall standardisation methods

We applied four different standardisation methods to obtain a single time series of monthly rainfall across all available gauges in the Mara for analysis:

## Arithmetic mean

$x_{n}=\frac{\sum_{b\in\mathfrak{B}_{n}} \dot{x}_{nb}}{B_{n}}$ (1)

where $\dot{x}_{nb}$ are the rainfall records for each month *n* = 0,…, *N*‒ 1 and where $\mathfrak{B}_{n}$ is the set of all the gauges that were active during month $n$. There are a total of *B_n_* gauges in the set. This simply calculates the arithmetic average of the total monthly rainfall across all rain gauges that were active in a given month and year.

## Mean-weighted average

$x_{n}=\frac{\sum_{b\in\mathfrak{B}_{n}} \dot{x}_{nb}\times\bar{x}_{b}}{\sum_{b\in\mathfrak{B}_{n}} \bar{x}_{b}}$ (2)

where $\bar{x}_{b}$ is the arithmetic mean of the rainfall values recorded at gauge $b$ in all months *n* = 0,…, *N*‒ 1. There are a total of *B* rain gauges. The other terms are defined as for Eq. 1. This calculates the average rainfall across all rain gauges that were active in a given month and year weighted by the mean of the rainfall records available across all months and years from the active gauges. The weight for each rain gauge is the overall average rainfall for that gauge.

## Gauge-adjusted mean

$x_{n}={\sum_{b\in\mathfrak{B}_{n}} (\frac{\dot{x}_{nb}}{\bar{x}_{b}}\times\frac{\sum_{b=1}^{B} \bar{x}_{b}}{B})}/{B_{n}}$ (3)

The total monthly rainfall from each rain gauge that was active in a given month and year is divided by the arithmetic mean of rainfall at that rain gauge across all months and years with records from that gauge. The resulting ratios were then multiplied by the overall mean of all the means derived from all available records of every individual gauge and divided by the number of active gauges. The total monthly rainfall for each rain gauge is thereby adjusted by the average rainfall of that gauge.

## Predictions from a generalized linear mixed model

$x_{n}=\frac{\sum_{n=1}^{B} \tilde{x}_{nb}}{B}$ (4)

where $\tilde{x}_{nb}$ are the best linear unbiased predictions (BLUPS) of rainfall values from a generalized linear mixed model for each month and rain gauge. Total monthly rainfall was modelled (S1 File) as a dependent variable with a date effect (a categorical variable with 603 levels) and a rain gauge random effect using a Tweedie error distribution and a power link function in the R-packages tweedie [174,175], MASS [176] and statmod [177]. This model also predicts rainfall for months and years with missing records and accounts for the imbalances in the set of active rain gauges during each month and year.

The monthly time series of rainfall were then summed to calculate the annual and seasonal rainfall components. The time series obtained with the different standardisation methods were very similar from 1965 until 2001, after which deviations between them increased (S1 Fig). Only two gauges were active after December 2003: Hyena Camp (January 2004-March 2015) and Serena Lodge (July 2008-December 2013). These gauges received the second (Serena Lodge) and third (Hyena Camp) highest overall mean monthly rainfall compared to the other gauges. Adjusting by the gauge mean (Eq. 3) performed best in controlling for the increase in rainfall due to the reduction in the number of recording gauges after 2003 (S1 Fig). We therefore used the time series of the gauge-adjusted means in subsequent analyses.

## References

173. Dunn PK, Smyth GK. Series evaluation of Tweedie exponential dispersion model densities. Statistics and Computing. 2005;15: 267–280.

174. Dunn PK, Smyth GK. Evaluation of Tweedie exponential dispersion model densities by Fourier inversion. Statistics and Computing. 2008;18: 73–86.

175. Venables WN, Ripley BD. Modern Applied Statistics with S. Fourth edition. New York, USA: Springer; 2002.

176. Giner G, Smyth GK. statmod: probability calculations for the inverse Gaussian distribution. R Journal. 2016;8: 339–351.
