# Supplementary material for: Rainfall trends and variation in the Maasai Mara ecosystem and their implications for animal population and biodiversity dynamics
Source: PLoS One. 2018 Sep 19;13(9):e0202814. doi: 10.1371/journal.pone.0202814 (PMC6145597; doi:10.1371/journal.pone.0202814)
Supplement: S2 Text — (DOCX) [file pone.0202814.s004.docx]

S2 Text. Modelling seasonal oscillations in rainfall

The monthly seasonal oscillations were modelled with trigonometric components


[1] as follows

$\gamma_{n}=\sum_{j=1}^{\left[ s/2 \right]} \gamma_{j,n},$ (1)

where *s* are the 12 months in each year and *j* = 1; 2,…, [*s*/2] is the index for the seasonal harmonics. The frequency of the sinusoids or harmonics *γ_j,n_* are *λ_j_ =*2*πj⁄s.* The sinusoids are specified by

$\left[ \begin{matrix} \gamma_{j,n} \\ \gamma_{j,n}^{*} \end{matrix} \right]=\left[ \begin{matrix} cos\lambda_{j} & sin\lambda_{j} \\ -sin\lambda_{j} & cos\lambda_{j} \end{matrix} \right]\left[ \begin{matrix} \gamma_{j, n-1} \\ \gamma_{j,n-1}^{*} \end{matrix} \right]+\left[ \begin{matrix} \varrho_{j,n} \\ \varrho_{j,n}^{*} \end{matrix} \right], {\varrho_{j,n}, \varrho}_{j,n}^{*}\sim i.i.d. N\left( 0,\sigma_{\varrho}^{2} \right),$ (2)

where the disturbances $\varrho_{j,n}$ and $\varrho_{j,n}^{*}$ are assumed to be i.i.d. normal deviates. If *s* is even, the equation for $\gamma_{s/{2,n}}^{*}$ is redundant and $\gamma_{s,2n}$ is given by

$\gamma_{s/2,n}=-\gamma_{s/2,n-1}+\varrho_{s/2,n}$. (3)

The autoregressive component $\dot{a}_{n}$ is defined as

$\dot{a}_{n}={\dot{\rho}\dot{a}}_{n-1}+\varsigma_{n},\varsigma_{n}\sim i.i.d. \left( 0,\sigma_{\varsigma}^{2} \right),$ (4)

in which $\dot{\rho}$ is the damping factor with the stochastic disturbance variance $\sigma_{\varsigma}^{2}$. The autoregressive component corresponds to a cycle with a frequency of 0 or *π*.

Reference

1. SAS Institute Inc. SAS/ETS® 14.1 user’s guide: the UCM procedure [software]. Cary, North Carolina, USA: SAS Institute Inc. 2015. Available from: https://support.sas.com/documentation/onlinedoc/ets/141/ucm.pdf
