# Supplementary material for: Rainfall trends and variation in the Maasai Mara ecosystem and their implications for animal population and biodiversity dynamics
Source: PLoS One. 2018 Sep 19;13(9):e0202814. doi: 10.1371/journal.pone.0202814 (PMC6145597; doi:10.1371/journal.pone.0202814)
Supplement: S3 Text — (DOCX) [file pone.0202814.s005.docx]

**S3 Text. Using wavelet analysis to detect changing periodicity in monthly rainfall oscillations**

The continuous wavelet transform of the de-seasonalized monthly rainfall values $\dot{x}_{n}$ is the convolution of $\dot{x}_{n}$ with a scaled (divided by *s^w^*) and translated (multiplied by (*n'*‒ *n*)*δt^w^*) version of the Morlet wavelet function *ψ*_0_(*κ*)


[1]:

$W_{n}\left( s^{w} \right)= \sum_{n^{'}= 0}^{N-1} \dot{x}_{n'}\psi^{*}\left[ \frac{(n^{'}-n)\delta t^{w}}{s^{w}} \right],$ (1)

where (*) signifies the complex conjugate, *ψ* is the normalized wavelet function derived from Eq. 11 with unit energy, *s^w^* is the wavelet scale, *n =*0,*…*, *N* ‒ 1 is the localized time index with *N* denoting the number of months in the time series and *δt^w^* are the time steps


[1]. Convolution in general terms is the integral of the product of two functions after one function has been reversed and shifted


[2], which for the wavelet transform translates into a sum of the products of the data series and the wavelet function.

Unit energy at each scale *s^w^* is satisfied if

$\hat{\psi} \left( s^{w}\omega_{k} \right)=\left( \frac{2\pi s^{w}}{\delta t^{w}} \right)^{1/2}\hat{\psi}_{0}\left( s^{w}\omega_{k} \right),$ (2)

where *k =*0,*…*, *N*‒ 1 is the frequency index


[1]. The convolution is done *N* times for each scale, thereby constructing a picture of how the amplitude of *W_n_*(*s^w^*) varies with time


[1]. The calculations were done in Fourier space


[1].

The wavelet power spectrum is defined as *|W_n_*(*s^w^*)*|*^2^


[1], where |.| indicates the Euclidean norm operator of the wavelet transform from Eq. 12. The bias-corrected power (i.e. reduced power at higher frequency fluctuations;


[3]) of the wavelet power spectrum was divided by the variance of the monthly rainfall values and plotted for each month and year to yield a graphical representation of the power of periodicity in rainfall oscillations over time. Normalizing with the variance expresses the power relative to white noise


[1].

The distribution for the local wavelet power spectrum at each time *n* and scale *s* is

$\frac{\left| W_{n} \right|^{2}}{\sigma^{2}}\Longrightarrow\frac{1}{2}P_{k}\chi_{2}^{2}$, (3)

where

$P_{k}=\frac{1-\alpha^{2}}{1+\alpha^{2}-2\alpha cos({2\pi k}/N)}$ (4)

is the red noise spectrum, *α* is the assumed lag ‒ 1 autocorrelation and *k =*0,*…*, *N*⁄2 is the frequency index


[1]. If *α =*0, Eq. 15 is equivalent to a white-noise spectrum.

The 95% confidence level contours of the normalized wavelet power spectra that are significantly higher than expected under the null hypothesis of a red noise AR(1) process were derived and plotted.

**References**

1. Torrence C, Compo GP. A practical guide to wavelet analysis. Bull Amer Meteor Soc. 1998;79: 61–78.

2. Shao J. Statistical functionals. In: Casella G, Fienberg S, Olkin I, editors. Mathematical statistics. New York, USA: Springer 2003. pp. 338–351.

3. Veleda D, Montagne R, Araujo M. Cross-wavelet bias corrected by normalizing scales. J Atmos Oceanic Technol. 2012;29: 1401–1408.
