# Supplementary material for: Rainfall trends and variation in the Maasai Mara ecosystem and their implications for animal population and biodiversity dynamics
Source: PLoS One. 2018 Sep 19;13(9):e0202814. doi: 10.1371/journal.pone.0202814 (PMC6145597; doi:10.1371/journal.pone.0202814)
Supplement: S4 Text — (DOCX) [file pone.0202814.s006.docx]

S4 Text. Threshold selection for estimating return levels of droughts and floods

The generalized Pareto distribution was used as an approximation to the distribution of excesses over a high threshold given by

$H\left( r \right)=1-\left[ 1+\varepsilon\left( \frac{r-u}{o_{u}} \right) \right]_{+}^{{-1}/\varepsilon},$ (8)

where *r =*($r_{0},\ldots,r_{T-1})$ is a vector of the annual and seasonal rainfall components, *u* is a high threshold, *r > u*, scale parameter *o_u_ >*0 (depending on threshold $u$) and shape parameter ‒ ∞ < ε < ∞


[1]. *H*(*r*) is only defined if the value inside the square bracket is positive.

We selected thresholds based on the stability of the scale and shape parameter estimates of the generalized extreme value distribution


[1] under the restriction of a minimum sample size of more than 15 threshold excesses. We progressively increased thresholds starting with the average of the annual and seasonal rainfall components up to the maximum possible threshold before the estimates of the scale and shape parameters became unstable or threshold excesses became 15 or less.

We also used the generalized Pareto distribution to approximate the distribution of extremely low annual and seasonal rainfall components after multiplying *u* and *r* with ‒ 1. The parameters were estimated by the maximum likelihood method.

The estimated return level of the annual and seasonal rainfall components *r* that is either exceeded by floods or not reached by droughts every *m* observations is

$\hat{r}_{m}= \left\{ \begin{matrix} u+ \frac{\hat{o}_{u}}{\hat{\varepsilon}} \left[ \left( m\hat{\zeta}_{u} \right)-1 \right] \text{if} \hat{\varepsilon}\neq0, \\ u+\hat{o}_{u} ln\left( m\hat{\zeta}_{u} \right) \text{if} \hat{\varepsilon}=0, \end{matrix} \right.$ (9)

where $\hat{\zeta}$*_u_* is the estimated probability of exceeding or not reaching the threshold *u* [1]. $\hat{\zeta}$*_u_* is obtained by dividing the total number of threshold excesses by the total number of years.

Reference

1. Gilleland E, Katz RW. Extremes 2.0: an extreme value analysis package in R. J Stat Softw. 2014;72: doi: 10.18637/jss.v072.i08.
