# Supplementary material for: Rainfall trends and variation in the Maasai Mara ecosystem and their implications for animal population and biodiversity dynamics
Source: PLoS One. 2018 Sep 19;13(9):e0202814. doi: 10.1371/journal.pone.0202814 (PMC6145597; doi:10.1371/journal.pone.0202814)
Supplement: S5 Text — (DOCX) [file pone.0202814.s007.docx]

S5 Text. Estimation of the runs and intervals estimator to detect multiannual persistence of droughts and floods

The runs estimator


[1] is given by

$\hat{\theta}_{l}\left( \hat{r}_{m};\dot{r} \right)= L^{-1}\left\{ 1+\sum_{a=1}^{L-1} I(E_{a}> \dot{r}) \right\},$ (1)

where $\dot{r}$ = 1 is the run length and *E_a_ = F_a+_*_1_*– F_a_* for *a =*1*,…,* *L*‒ 1 are the observed number of years between the droughts that exceeded or floods that did not reach the return levels $\hat{r}_{m}$ estimated from Eq. 9. Let 1 *≤ F*_1_ *< … < F_L_ ≤ l* be the exceedance times. If $\hat{\theta}_{l}\left( \hat{r}_{m} \right)< 1$, then droughts and floods cluster over consecutive years.

The intervals estimator


[1] is given by

$\breve{\theta}_{l}\left( \hat{r}_{m} \right)=\left\{ \begin{aligned} 1\wedge\tilde{\theta}_{l}\left( \hat{r}_{m} \right) if max \left\{ E_{a}:1\leq a\leq L-1 \right\}\leq2, \\ 1\wedge\tilde{\theta}_{l}^{*}\left( \hat{r}_{m} \right) if max \left\{ E_{a}:1\leq a\leq L-1 \right\}>2, \end{aligned} \right.$ (2)

where

$\tilde{\theta}_{l}\left( \hat{r}_{m} \right)=\frac{2\left( \sum_{a=1}^{L-1} E_{a} \right)^{2}}{\left( L-1 \right)\sum_{a=1}^{L-1} E_{a}^{2}} ,$ (3)

and

$\tilde{\theta}_{l}^{*}\left( \hat{r}_{m} \right)=\frac{2\left\{ \sum_{a=1}^{L-1} \left( E_{a}-1 \right) \right\}^{2}}{(L-1)\sum_{a=1}^{L-1} \left( E_{a}-1 \right)(E_{a}-2)} .$ (4)

If $\breve{\theta}_{l}\left( \hat{r}_{m} \right)< 1$, then the drought and flood years tend to cluster in time.

**Reference**

1. Ferro CAT, Segers J. Inference for clusters of extreme values. J R Stat Soc Series B Stat Methodol. 2003;65: 545–556.
