# Supplementary material for: Rainfall trends and variation in the Maasai Mara ecosystem and their implications for animal population and biodiversity dynamics
Source: PLoS One. 2018 Sep 19;13(9):e0202814. doi: 10.1371/journal.pone.0202814 (PMC6145597; doi:10.1371/journal.pone.0202814)
Supplement: S6 Text — (DOCX) [file pone.0202814.s008.docx]

S6 Text. Quantile regression to analyse trends in the severity of droughts and floods

Based on the linear model

$R_{t}=y_{t}\beta+v_{i}, v_{i}\sim i.i.d. F_{v},$ (1)

where *y_t_* is the sequence of years and *R_t_* are now either the annual or the seasonal rainfall components for each year *t*, the *q*-dimensional regression quantiles *θ* are defined as solutions to


[1]

$R_{\theta}\left( \hat{\beta} \right)=\begin{matrix} argmin \\ \hat{\beta}\in R^{q} \end{matrix}\sum_{t=0}^{T-1} \rho_{\theta}\left( r_{t}-y_{t}\hat{\beta} \right)$, (2)

where *r_t_* are now the *q*-component annual or seasonal rainfall values for year *t*, where *θ ϵ*(0,1) and

$\rho_{\theta}(v)= \left\{ \begin{matrix} \theta v \mathrm{if} v\geq0, \\ (\theta-1)v \mathrm{if} v<0. \end{matrix} \right.$ (3)

An algorithmic linear programming method was used to minimize the weighted sum of absolute residuals according to Koenker and D’Orey


[1,2].

References

1. Koenker RW, D’Orey V. Algorithm AS 229: computing regression quantiles. Appl Stat. 1987;36: 383–393.

2. Koenker R, D’Orey V. Remark AS R92: a remark on algorithm AS 229: computing dual regression quantiles and regression rank scores. Appl Stat. 1994;43: 410–414.
