# Supplementary figures and images for: Rainfall trends and variation in the Maasai Mara ecosystem and their implications for animal population and biodiversity dynamics
Source: PLoS One. 2018 Sep 19;13(9):e0202814. doi: 10.1371/journal.pone.0202814 (PMC6145597; doi:10.1371/journal.pone.0202814)

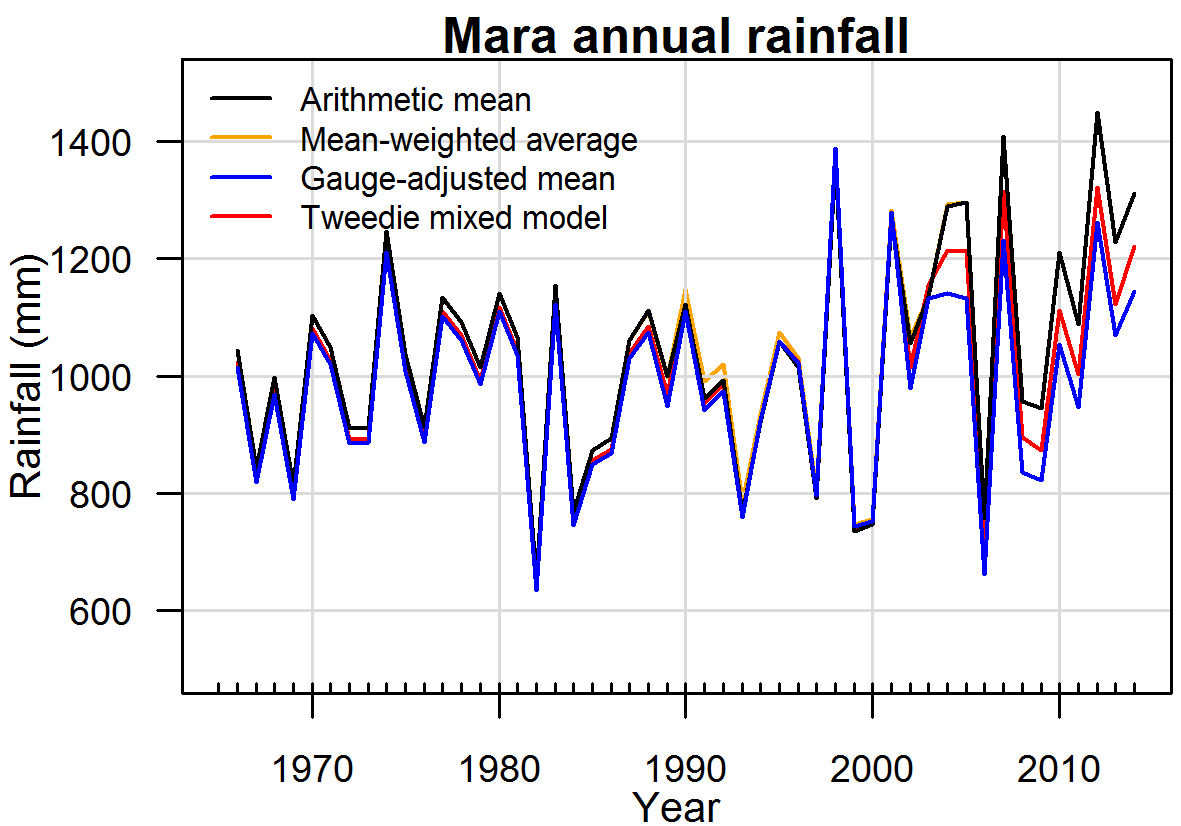

Supplement: S1 Fig — Four different standardisation methods were applied: arithmetic mean (black line), mean-weighted average (orange line), gauge-adjusted mean (blue line) and Best Linear Unbiased Predictions (BLUPS) from a generalized linear mixed model with Tweedie error distribution and a log link function (red line). (TIFF) [file pone.0202814.s011.tiff]

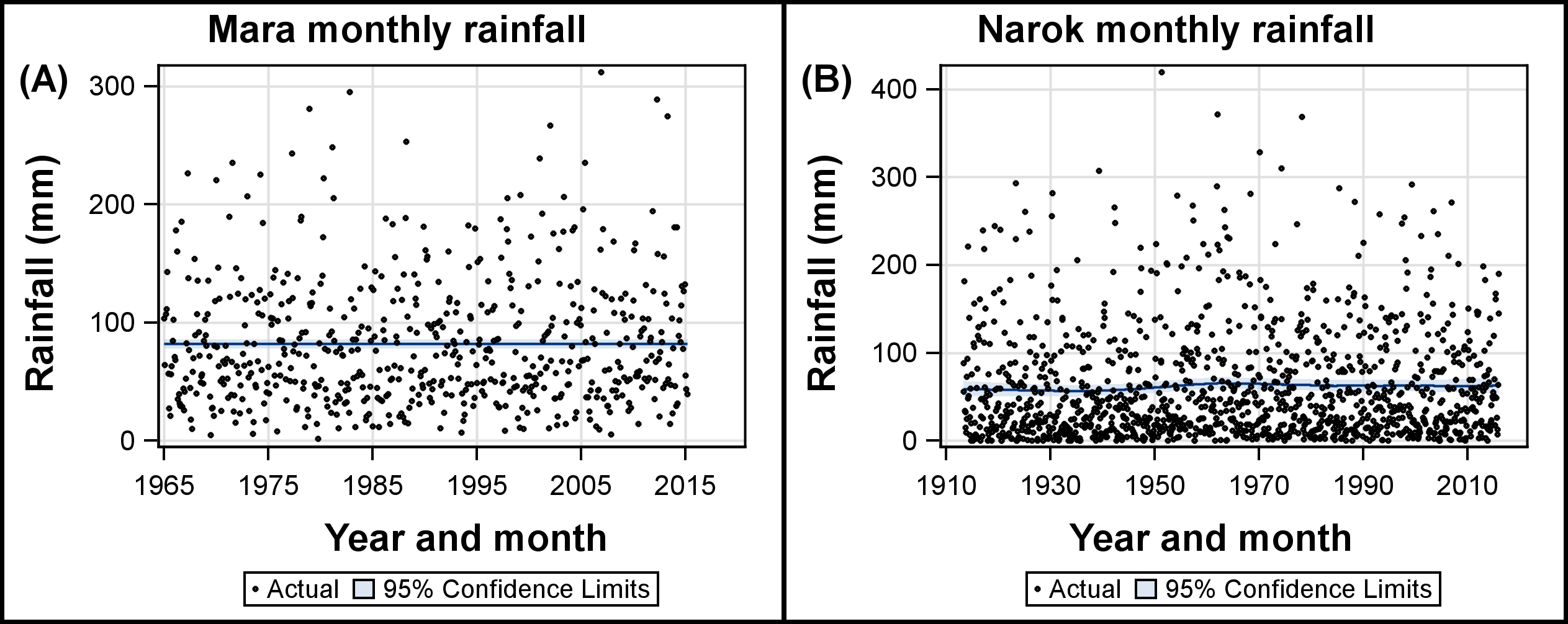

Supplement: S2 Fig — (A) Rainfall recordings in the Mara were derived from 15 gauges (Eq 3 in S1 Text) recording in the Mara during 1965–2014 and in (B) Narok Town in Kenya during 1913–2015. Significant components are marked in bold-faced font. (TIF) [file pone.0202814.s012.tif]

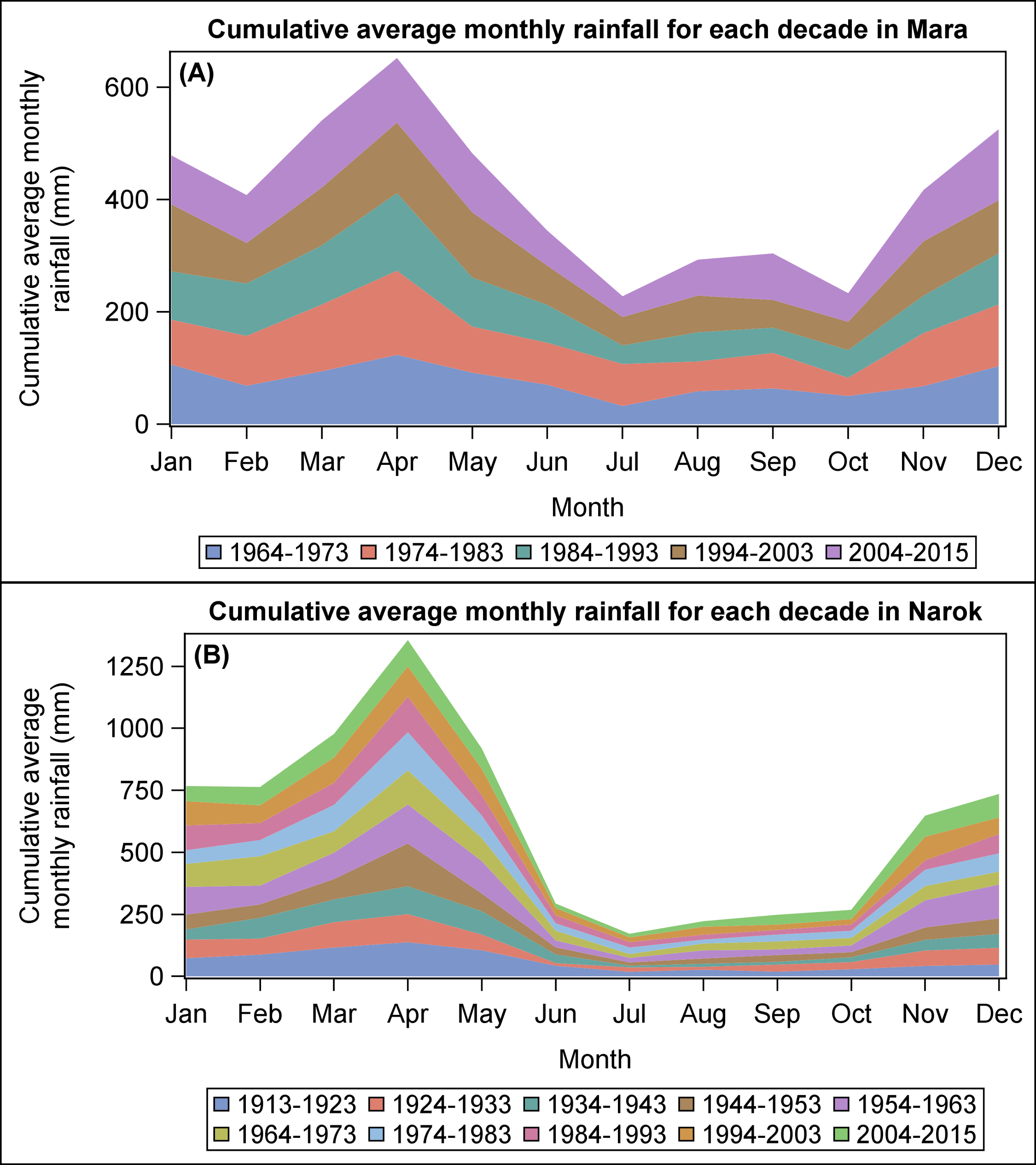

Supplement: S3 Fig — (A) Rainfall in the Mara during 1965–2014. (B) Rainfall in Narok Town in Kenya during 1913–2015. (TIF) [file pone.0202814.s013.tif]

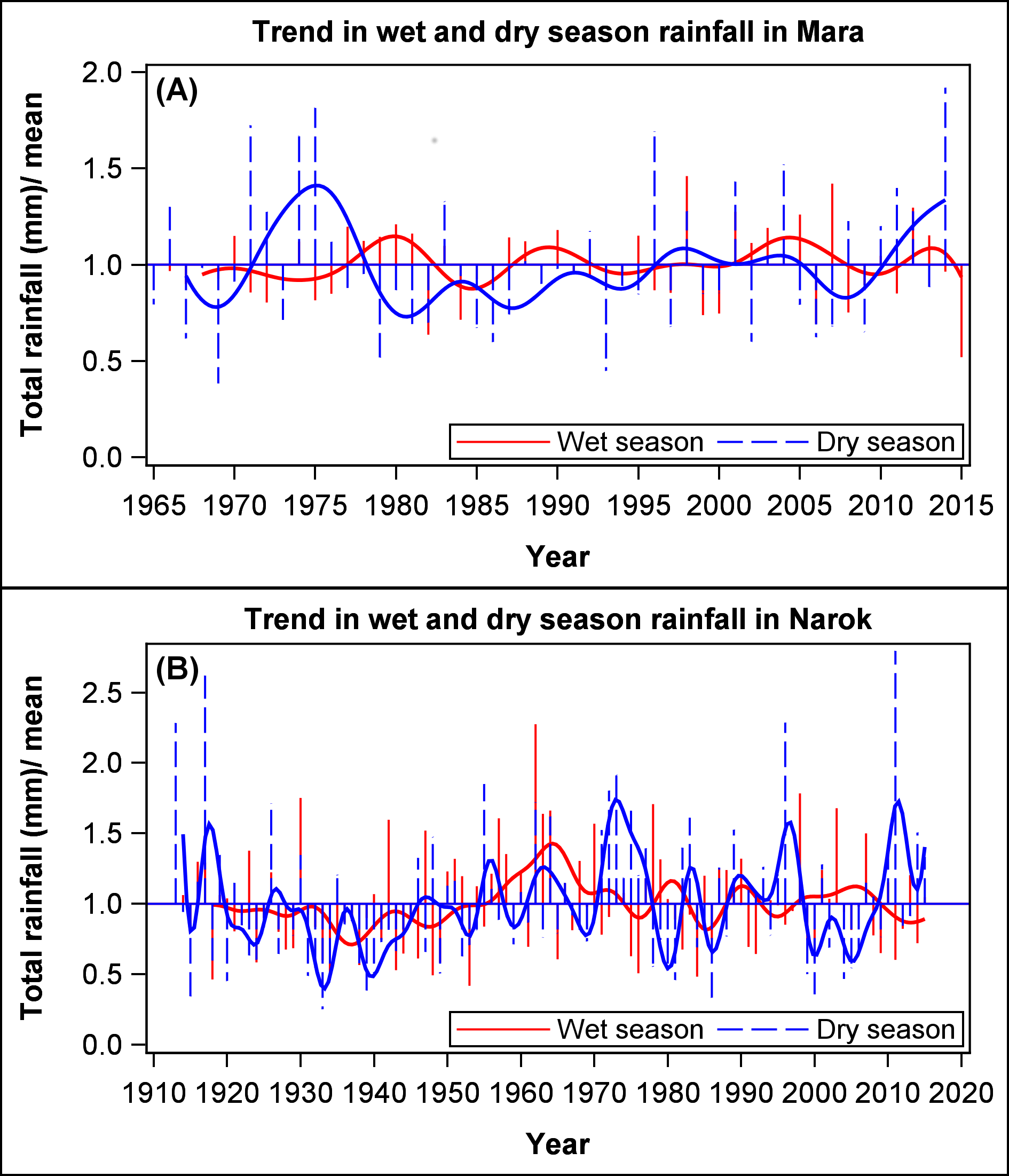

Supplement: S4 Fig — The vertical needles are the standardized deviates and the solid curves are the 3-year (Mara), 5-year (wet season of Narok Town) and 2-year (dry season of Narok Town) moving averages. (A) Rainfall recordings in the Mara were derived from 15 gauges (Eq 3 in S1 Text) during 1966–2014 (dry season: 1965–2014). (B) Rainfall in Narok Town in Kenya was recorded during 1914–2015 (dry season: 1913–2015). The wet season and dry season rainfall components were summed from the monthly rainfall records. (TIF) [file pone.0202814.s014.tif]

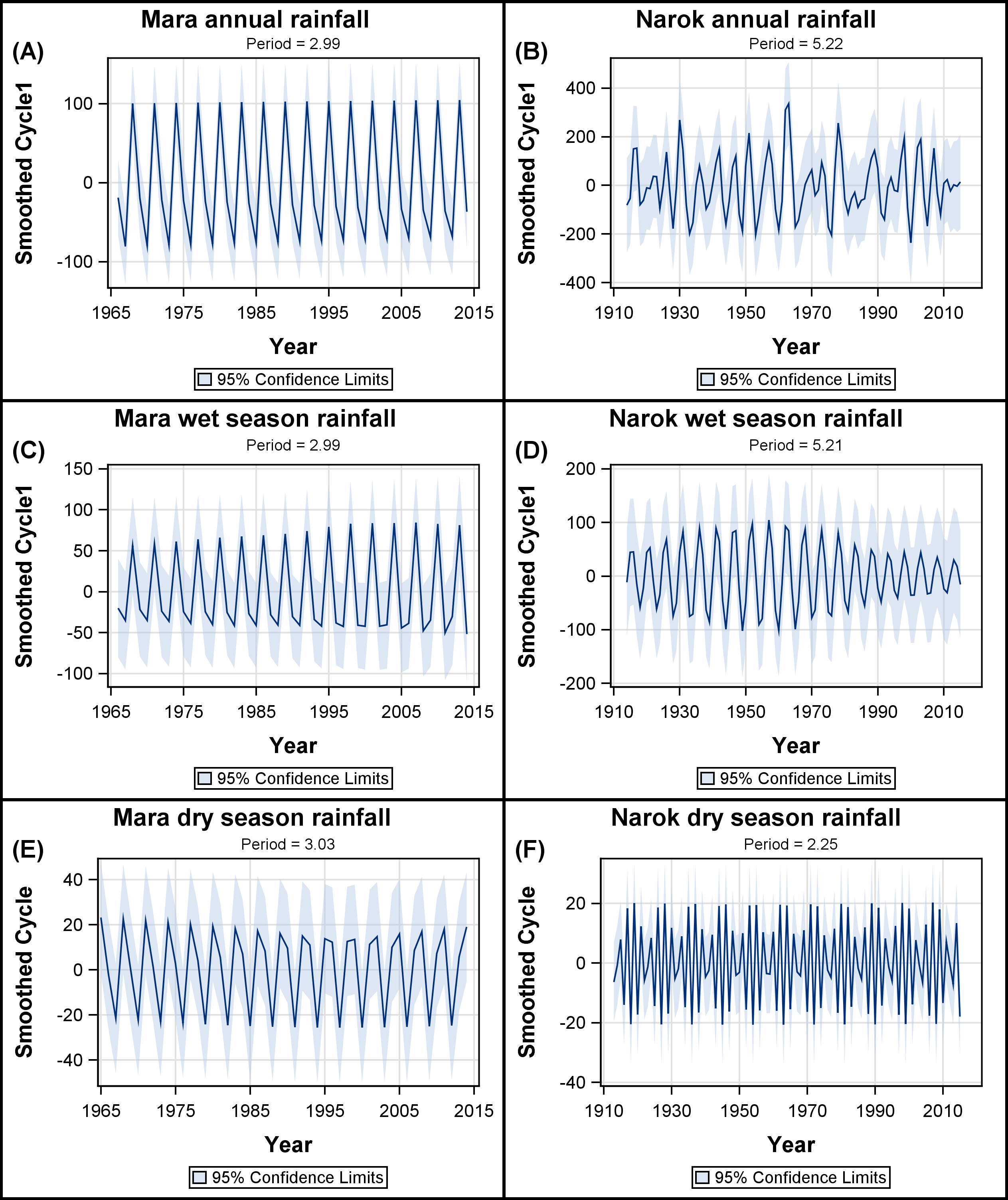

Supplement: S5 Fig — (A, C, D) Rainfall recordings in the Mara were derived from 15 gauges (Eq 3 in S1 Text) during 1966–2014 (dry season: 1965–2014). (B, D, F) Rainfall in Narok Town in Kenya was recorded during 1914–2015 (dry season: 1913–2015). The (A, B) annual, (C, D) wet season and (E, F) dry season rainfall components were summed from the monthly rainfall records. (TIF) [file pone.0202814.s015.tif]

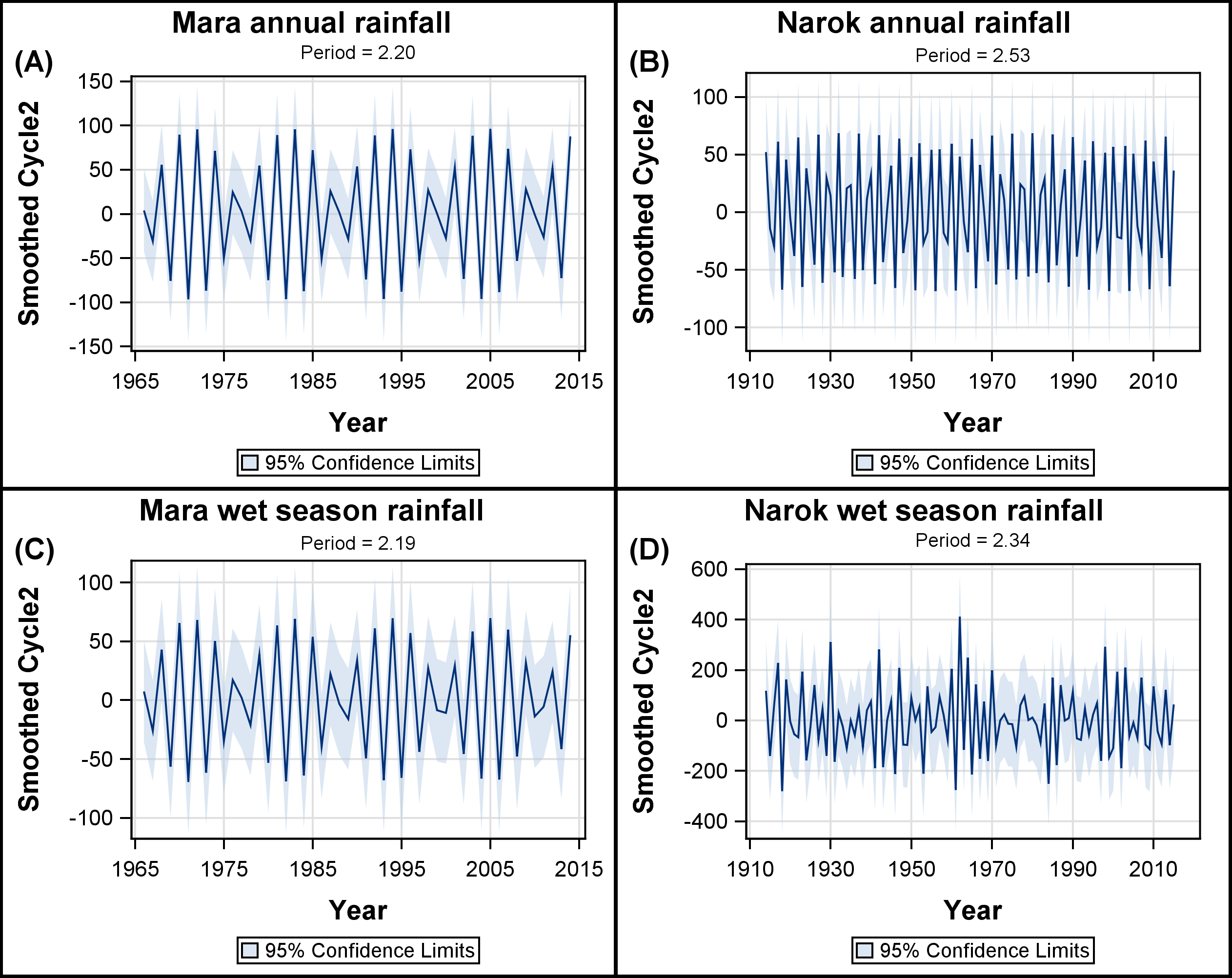

Supplement: S6 Fig — (A, C) Rainfall recordings in the Mara were derived from 15 gauges (Eq 3 in S1 Text) during 1966–2014 (dry season: 1965–2014). (B, D) Rainfall in Narok Town in Kenya was recorded during 1914–2015 (dry season: 1913–2015). The (A, B) annual and (C, D) wet season rainfall components were summed from the monthly rainfall records. (TIF) [file pone.0202814.s016.tif]

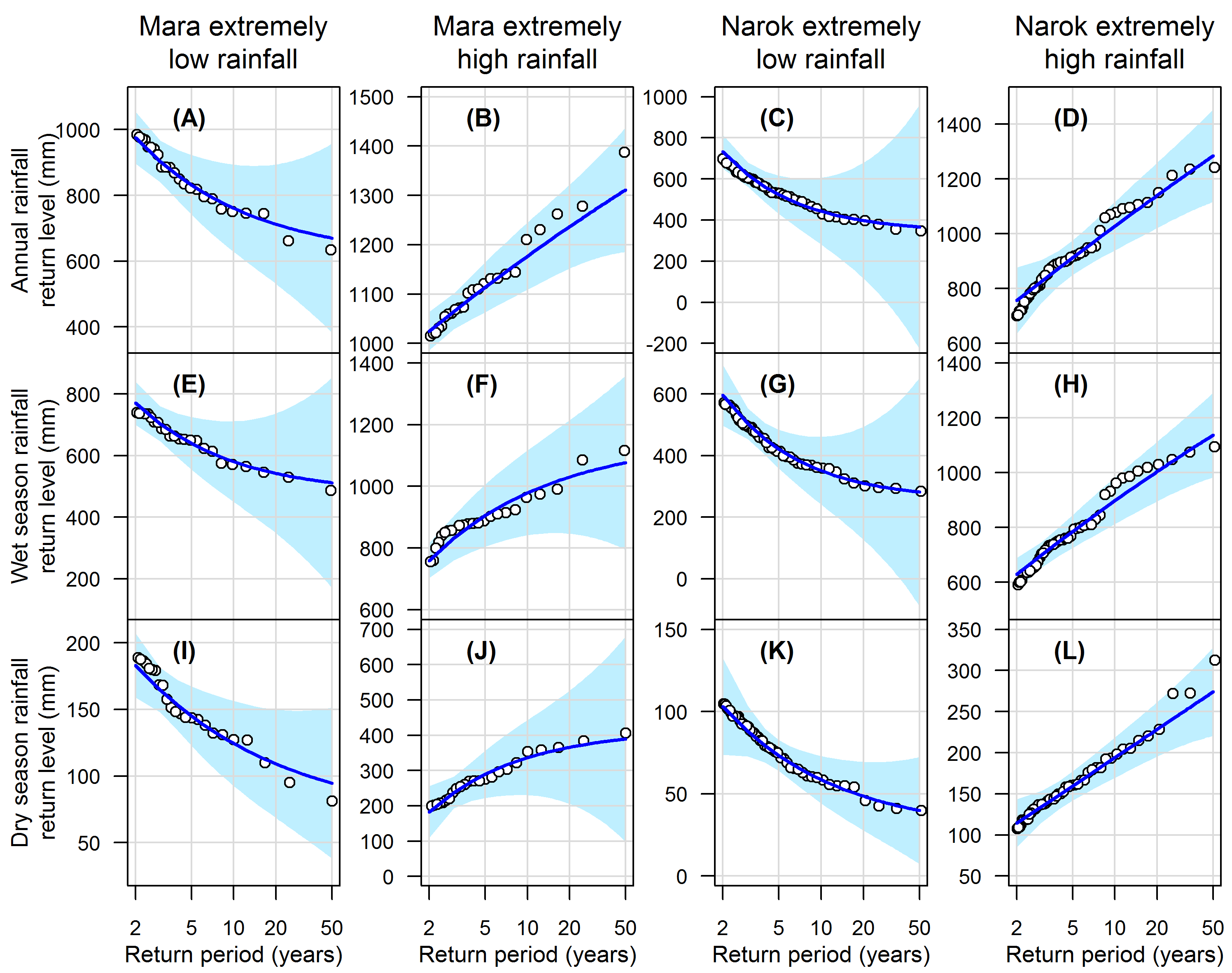

Supplement: S7 Fig — (A, B, E, F, I, J) Rainfall recordings in the Mara were derived from 15 gauges (Eq 3 in S1 Text) during 1966–2014 (dry season: 1965–2014). (C, D, G, H, K, L) Rainfall in Narok Town in Kenya was recorded during 1914–2015 (dry season: 1913–2015). The (A, B, C, D) annual, (E, F, G, H) wet season and (I, J, K, L) dry season rainfall components were summed from the monthly rainfall records. Blue polygons are the 95% normal approximate confidence bands. (TIFF) [file pone.0202814.s017.tiff]
